# Assembling the Community-Scale Discoverable Human Proteome

**DOI:** 10.1016/j.cels.2018.08.004

**Published:** 2018-08-29

**Authors:** Mingxun Wang, Jian Wang, Jeremy Carver, Benjamin S. Pullman, Seong Won Cha, Nuno Bandeira

**Affiliations:** 1 Center for Computational Mass Spectrometry and Department of Computer Science and Engineering, University of California, San Diego, La Jolla, San Diego, CA 92093, USA; 2 Electrical and Computer Engineering, University of California, San Diego, La Jolla, San Diego, CA 92093, USA; 3 Skaggs School of Pharmacy and Pharmaceutical Sciences, University of California, San Diego, La Jolla, CA 92093, USA; 4 Lead Contact

## Abstract

The increasing throughput and sharing of proteomics mass spectrometry data have now yielded over one-third of a million public mass spectrometry runs. However, these discoveries are not continuously aggregated in an open and error-controlled manner, which limits their utility. To facilitate the reusability of these data, we built the MassIVE Knowledge Base (MassIVE-KB), a community-wide, continuously updating knowledge base that aggregates proteomics mass spectrometry discoveries into an open reusable format with full provenance information for community scrutiny. Reusing >31 TB of public human data stored in a mass spectrometry interactive virtual environment (MassIVE), the MassIVE-KB contains >2.1 million precursors from 19,610 proteins (48% larger than before; 97% of the total) and doubles proteome coverage to 6 million amino acids (54% of the proteome) with strict library-scale false discovery controls, thereby providing evidence for 430 proteins for which sufficient protein-level evidence was previously missing. Furthermore, MassIVE-KB can inform experimental design, helps identify and quantify new data, and provides tools for community construction of specialized spectral libraries.

## In Brief

Wang et al. introduce MassIVE-KB, a program designed to distill the entire community’s mass spectrometry data into reusable spectral library resources. As a result, the statistically-significant discovery of a peptide or protein in a single researcher’s data will thus be made available to the whole community to support its identification (in shotgun experiments) or quantitative detection (in targeted experiments) in all future analyses.

## Graphical Abstract

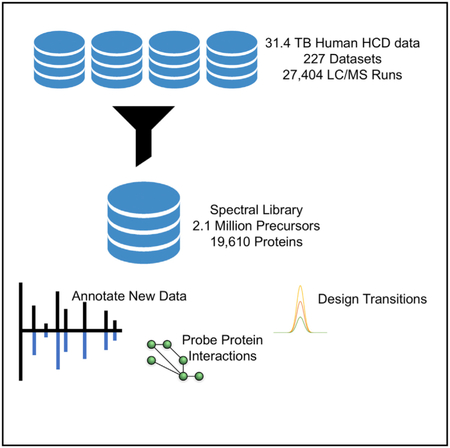

## INTRODUCTION

The community-scale verifiable mapping of the human proteome is essential to the characterization of biological processes and their relationship to health and disease. While several large-scale proteomics efforts (Kim et al., 2014; Rosenberger et al., 2014; Wilhelm et al., 2014) have endeavored to construct draft maps of the human proteome, the limited extent of their coverage shows that it remains impractical for a single study to be fully comprehensive, especially as increasing volumes of proteomics data are continuously made available in the public domain covering a wide diversity of tissues, cell types, and experimental conditions. Only by leveraging the breadth and depth of community-scale datasets can we systematically derive associations of peptide/protein observations to metadata charting their occurrence and expression profiles. Most importantly, any comprehensive map of the human proteome must include detailed provenance for all observations (to enable community scrutiny) and should be delivered as a verifiable reusable collection of reference spectra (i.e., a spectral library) to support biological discovery through detection and quantification in the new shotgun and targeted proteomics experiments.

These goals have motivated the construction of large-scale spectral libraries such as the PeptideAtlas (Farrah et al., 2013), National Institute of Standards and Technology (NIST) (Dong et al., 2014), and Global Proteome Machine Database (GPMDB) (Craig et al., 2006), but unfortunately these were mostly not designed to scale automatically with the growing availability of public human data (though some have been updated as new data become available) and do not include a complete provenance record of how they were derived or do not include a detailed description of library-level controls for false discovery rates (FDRs). As such, the requirement of manual intervention to update these resources has resulted in collections with limited coverage and lacking detailed provenance records linking library entries to the raw data, searches, and software used to construct the libraries. The absence of provenance records and algorithmic details is especially relevant, as the field evolves to require more stringent criteria for controlling FDRs at multiple levels of proteomics discovery.

**Figure 1.**
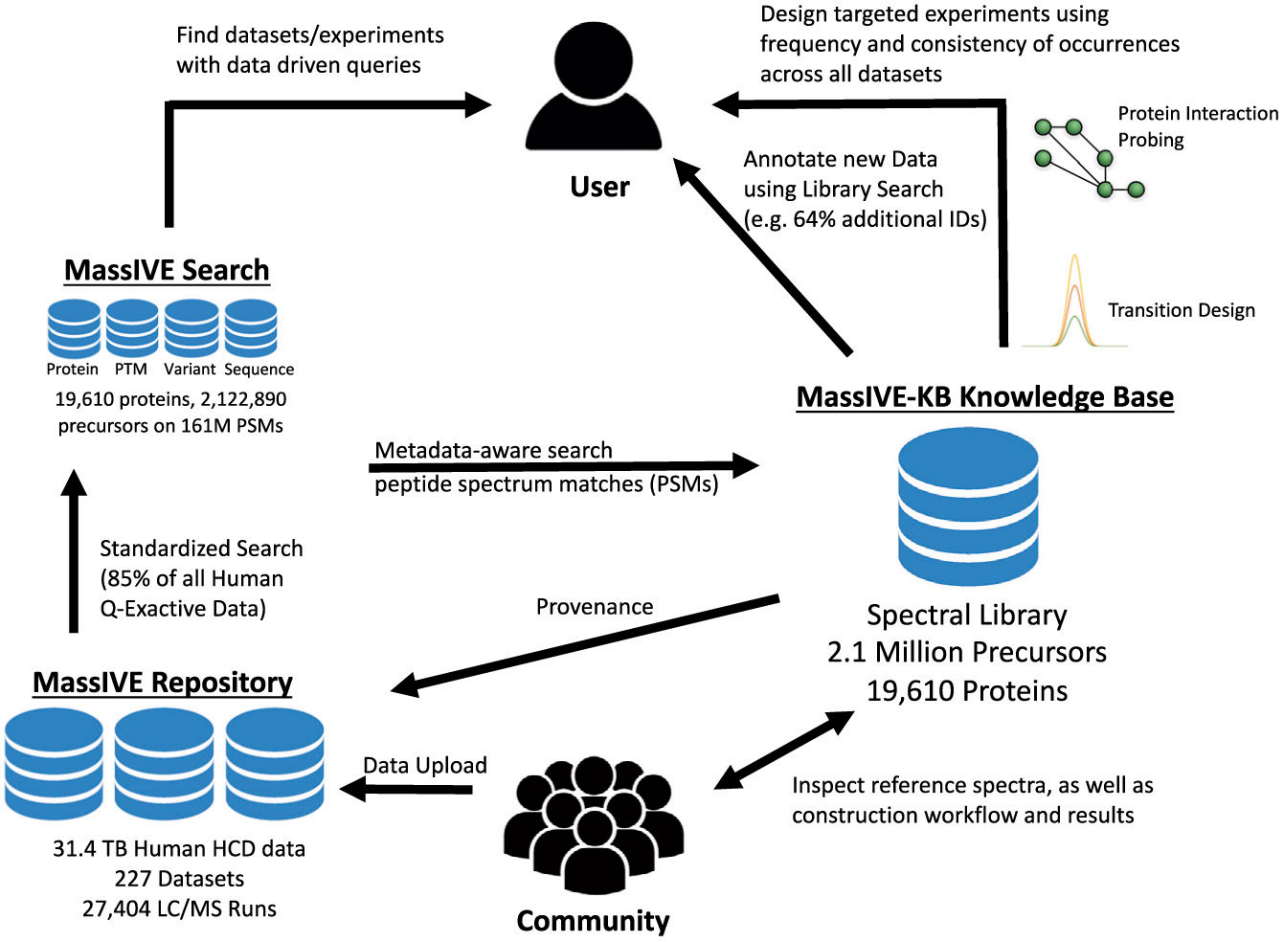
Overview of the MassIVE-KB Representation of the interactions between the proteomics community, public datasets, MassIVE Search, and MassIVE-KB derived from over 31.4 TB of MS data with 658 million MS/MS spectra yielding 191 million PSMs aggregated into a spectral library of 2.1 million precursors; new datasets will be incrementally added to MassIVE-KB upon deposition in ProteomeXchange repositories. This platform promotes reutilization of proteomics MS big data in three major ways: (1) increasing identifications in new liquid chromatography-tandem mass spectrometry (LC-MS/MS) data by spectral library search against a community-scale reference collection, (2) validation of extraordinary results against reference library spectra and all identified spectra found in all public data, and (3) supporting experimental design with reference spectra and detailed records of community-wide occurrences (e.g., designing targeted proteomics experiment). Furthermore, all entries in MassIVE-KB are supported by open provenance records that can be scrutinized by the community to inspect the series of events that led to specific identifications and reference spectra.

**Figure 2.**
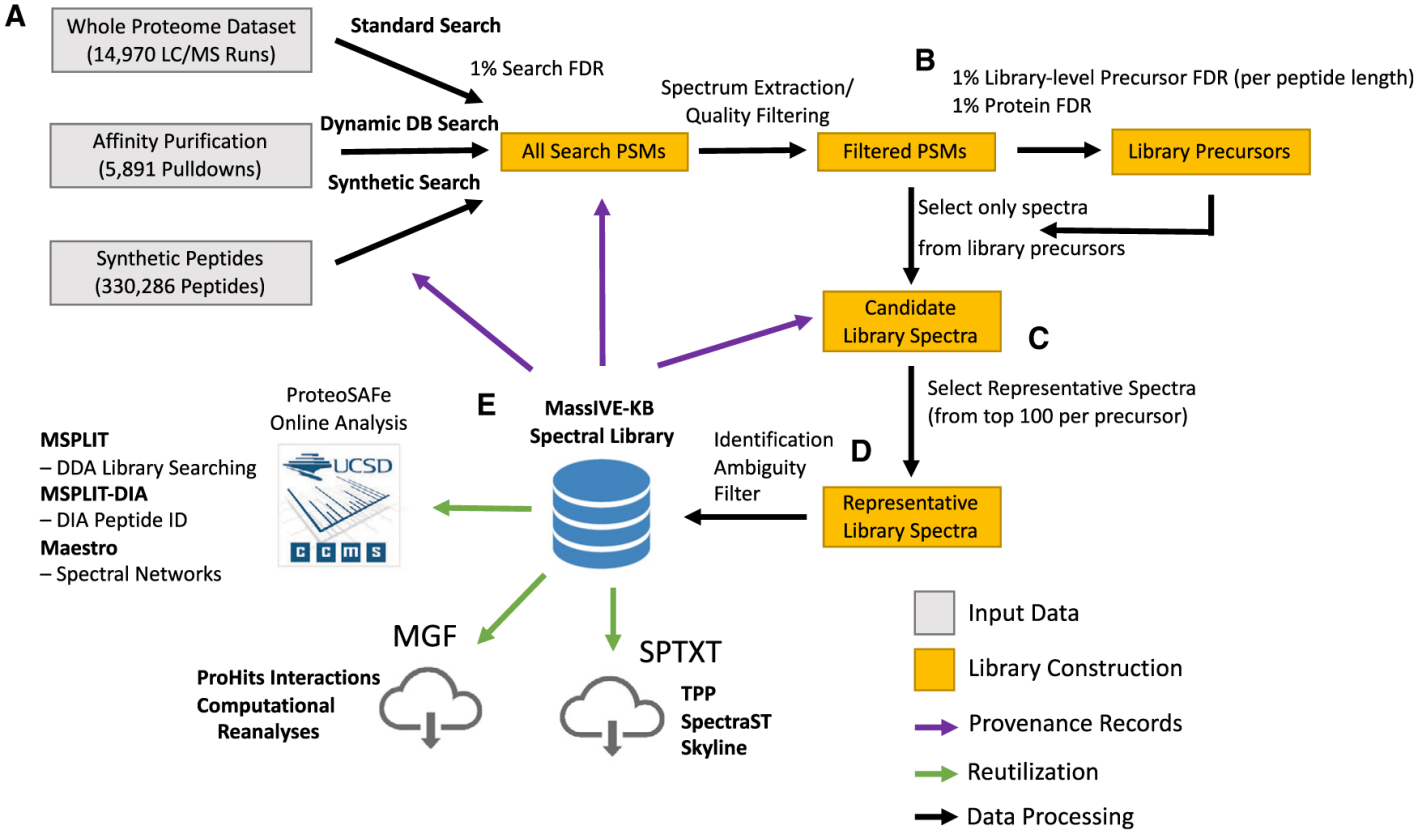
Step-by-Step Overview of the Spectral Library Construction Procedure (A)Datasets of varying complexity are first searched with workflows designed to appropriately capture the effective peptide search space defined by experimental metadata; false discovery rates (FDRs) are controlled per search, and PSMs are aggregated together and filtered by spectrum quality. (B)Precursor-level FDR is applied to filter candidate spectra for inclusion in the library. (C)The most similar spectrum to all other replicates is chosen as each precursor’s representative spectrum and (D) ambiguously identified spectra mapping to more than one peptide sequence are removed. (E) The MassIVE-KB spectral library can be readily applied to new searches at the Center for Computational Mass Spectrometry’s online workflow engine for data-dependent acquisition (DDA) library searching (MSPLIT), DIA peptide identification (MSPLIT-DIA), and spectral networks analysis (Maestro). In addition, MassIVE-KB can also be consulted online and is freely available for download in formats compatible with popular third-party tools such as the Trans-Proteomics Pipeline (Deutsch et al., 2010), SpectraST (Lam et al., 2007), and Skyline (MacLean et al., 2010).

We propose a new approach and the mass spectrometry interactive virtual environment knowledge base (MassIVE-KB) for the reusable aggregation of community-scale detection of peptides and proteins observations. Our approach is composed of multiple steps designed to scale to all public human proteomics data (Figures 1 and 2). First, our approach is designed to independently search new data as they become available, using only open-source algorithms and search procedures. Second, we build on advanced statistical controls for peptide identifications to automatically adjust the effective search space for each dataset, thereby capturing the statistical advantage of simpler samples while retaining stringency over a range of sample complexity. Third, we impose additional statistical controls for the community-scale aggregation of peptide identifications, thereby avoiding the uncontrolled accumulation of false discoveries. Fourth, our online-algorithms approach guarantees that incremental extension of the library yields the same results as *ab initio* reconstruction, thereby eliminating the need for time-consuming reprocessing when integrating newly available data. Fifth, all identifications in the library are supported by detailed provenance records linking each identification to not only the source raw data but also of the details of the search procedure used to derive the results (Figure 2; Data S1). Sixth, MassIVE-KB is readily reusable with existing search tools, including both traditional data-dependent (mixture-spectrum partitioning using libraries of identified tandem mass spectra [MSPLIT]) (Wang et al., 2010) and more recent data-independent (MSPLIT-DIA) (Wang et al., 2015) acquisition modes, as well as other popular tools such as Skyline (MacLean et al., 2010), SpectraST (Lam et al., 2007), and ProHits (Liu et al., 2010). Lastly, MassIVE-KB also delivers a publicly accessible pipeline that enables the community to create their own spectral libraries (see STAR Methods: Documentation).

## RESULTS

### Aggregation/Search of Human Proteomics Data

The initial release of MassIVE-KB focuses on the currently most popular dissociation mode in public proteomics data—higher energy collisional dissociation (HCD) Orbitrap, which covers 75.6% of all public human data deposited in the last 18 months. The MassIVE-KB human HCD spectral library is built from public human datasets (Table S1) available at the Center for Computational Mass Spectrometry’s (CCMS) MassIVE data repository (a full member of the ProteomeXchange consortium [Deutsch et al., 2017]). All datasets are stored in MassIVE to enable reanalysis and public access to provenance records, including several datasets that were originally deposited in PRoteomics IDEntifications (PRIDE) (Vizcaino et al., 2013) and are now mirrored at MassIVE. All together, these datasets contain 658 million tandem mass spectrometry (MS/MS) spectra from 27,404 liquid chromatography-tandem mass spectrometry (LC/MS) runs in 227 datasets totaling 31.4 terabytes (TB) (~85% of all public Human HCD Q-Exactive data). These data are grouped into three broad experimental categories: (1) whole proteome lysate analysis, (2) limited protein purification (e.g., affinity purification [AP] mass spectrometry [MS] [Huttlin et al., 2017]), and (3) synthetic peptide pools (Zolg et al., 2017) (Figure 2). This diversity of sample complexity was explicitly modeled in our search strategies by defining the confidence of a spectrum identification using both the peptide-spectrum match (PSM) score and its significance in relation to the search space of each sample (as determined by the experimental protocols). By explicitly modeling each search space, our approach is able to reflect the higher confidence in peptide identifications from synthetic peptides and, correspondingly, the lower confidence in identifications from whole-proteome cell lysates. By searching these data with the open-source database search tool MSGF+ (Kim and Pevzner, 2014) and using the proper adjustment for the various search spaces (Figures S1A and S1B; STAR Methods: Database p Value Calculation, Dynamic Search Space Adjustment for Proteome Limited Data, and ProteomeTools Synthetic Searching). Our procedure improved the significance of database p values by up to three orders of magnitude (Figure S1C).

FDRs in MassIVE-KB are initially controlled at the level of the individual dataset searches (1% PSM-level FDR estimated by target-decoy approach—TDA [Elias and Gygi, 2007]) but must also be controlled at the aggregate level in the final spectral library to avoid an otherwise unacceptable accumulation of errors into a library-scale 28% precursor-level FDR (which would result if the dataset-level search results were naively merged without any additional FDR controls). MassIVE-KB requirements for global precursor error controls are different from the current library resources; for example, PeptideAtlas (Farrah et al., 2013) applies PSM-level FDR controls at a per-dataset level and protein FDR globally, but no explicit controls were described to assess the precursor FDR used for library creation. Similarly, GPMDB (Craig et al., 2006) relies on an expectation value for each peptide, but there is no explicit FDR calculation showing whether the threshold needs to be adjusted when aggregating many searches into a single set of identifications. NIST’s libraries (Dong et al., 2014) also do not provide guarantees for library-level FDRs at the precursor, peptide, or protein level.

**Figure 3.**
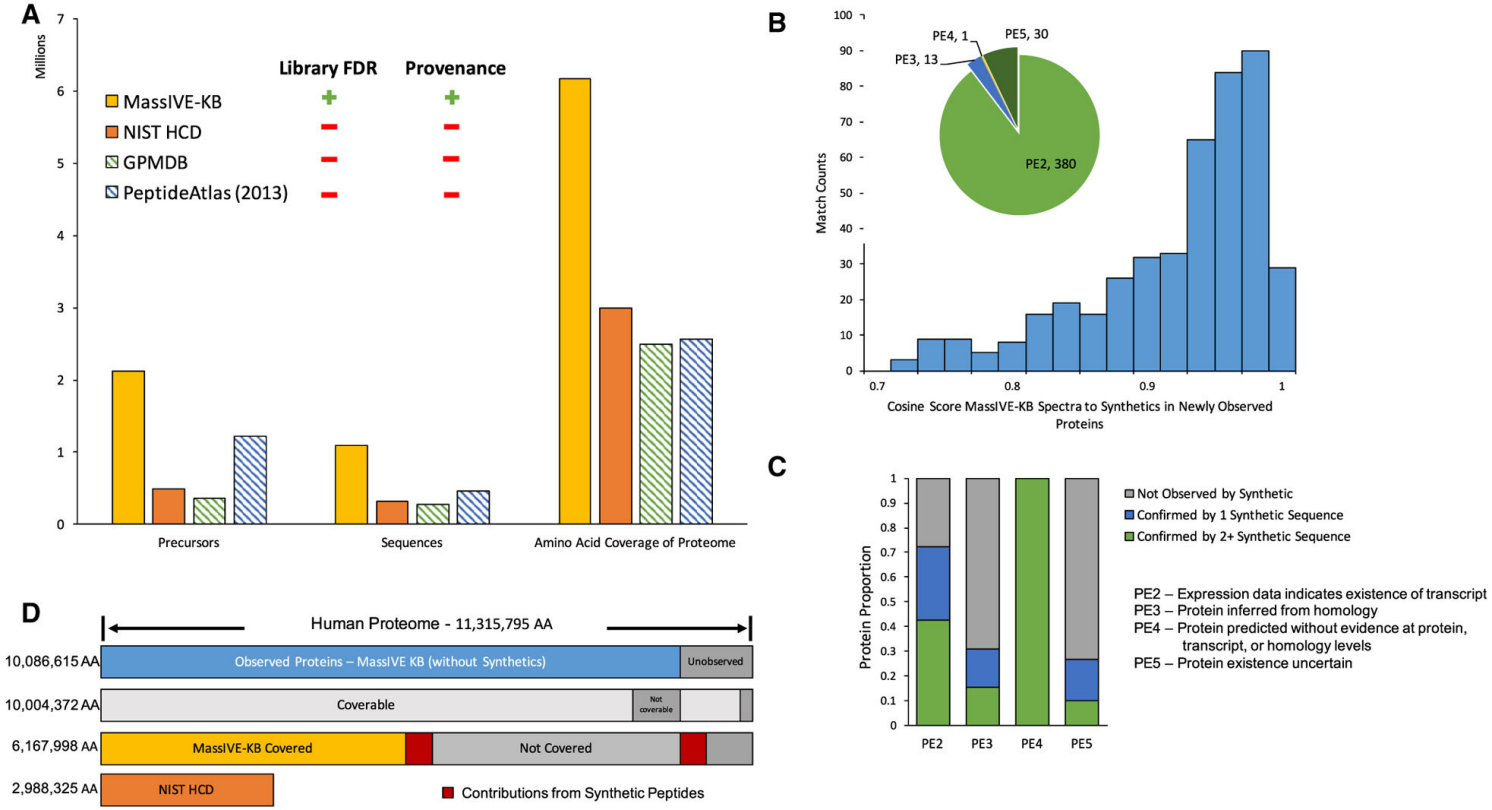
MassIVE-KB Coverage of the Human Proteome Reveals Hundreds of Novel Proteins (A) The MassIVE-KB HCD spectral library substantially expands over NIST’s HCD library (the largest comparable library) by including spectra from 4.3× more precursors from 3.4× more peptide sequences covering over twice as many amino acids in the human proteome. Furthermore, unlikeother available spectral library resources, MassIVE-KB explicitly controls library-level FDR at the precursor and protein levels, as well as provides comprehensive provenance information for every single library spectrum. (B)MassIVE-KB enabled the detection of 430 novel proteins—neXtProt PE2-5 broken down in pie chart insert, PE legend in part (C)–with at least two nonoverlapping sequences (stricter requirements than the standard HUPO identification guidelines [Deutsch et al., 2016]); the majority of these proteins were already observed with transcriptomics evidence (PE2, 380 proteins). All MassIVE-KB peptides uniquely mapped to novel proteins matched Proteome Tools synthetic peptides with highly correlated fragmentation patterns (0.93 median cosine) for all sequences included in their set. (C)ProteomeTools synthetic peptides fully confirmed 291 MassIVE-KB novel proteins (162 proteins with 2+ sequences). (D)Even with the marked MassIVE-KB gain in coverage of the human proteome, there remain 4.1 million “coverable” amino acids in fully tryptic sequences with ≤40 aminoacids; 3.4 million of these uncovered amino acids regions are in proteins that have been observed in non-synthetic data(i.e., missing coverage on known proteins) while 695 thousand amino acids remain uncovered in proteins that have not been observed with unique peptides from non-synthetic data.

Searching the human HCD data generated 191,152,777 PSMs. Synthetic peptide pool PSMs were filtered to 0% empirical FDR in each individual search (see STAR Methods: ProteomeTools Synthetic Searching) and at most 1% PSM level FDR in other searches (see STAR Methods: Single Pass Search; STAR Methods: Dynamic Search Space Adjustment Search; and STAR Methods: Dynamic Search Space Adjustment for Proteome Limited Data). All search results were aggregated and were globally controlled to 1% local precursor FDR (Elias and Gygi, 2007) per peptide length and 1% protein-level FDR (Savitski et al., 2015). After applying spectrum quality (see STAR Methods: Spectra Extraction/Filtering), precursor FDR, and protein FDR filters, we were left with 2,176,235 precursors at an estimated 0.1% global precursor FDR (Figure 3A; STAR Methods: Global Error Rate Controls for Spectral Library).

### Construction and Spectrum Quality of Peptide Spectral Library

For all the replicate MS/MS spectra of a precursor, we must select a single exemplar spectrum that exhibits the best evidence of the precursor to be reused as a reference for the community. This representative library spectrum was chosen from a truncated set of 100 PSMs per precursor from the original search results (see STAR Methods: MassIVE-KB Spectral Library Precursor Selection), totaling 30,160,134 PSMs (uniformly 0% q value in each PSM’s original search). The representative library spectrum was selected to be the most similar spectrum to all others from the same precursor (Figures S2A, S2B, and S3B). Of the representatives selected, 1,340,043 (62%) came from general proteomics data, 647,797 (30%) came from AP, and 188,395 (9%) came from synthetic peptide pools, demonstrating the complementarity of these data sources and proteomics groups (Table 1). To avoid potentially ambiguous identification of MS/MS spectra to insufficiently distinguishable peptides, all library spectra were re-searched with MSGF+ (Kim and Pevzner, 2014) against the reference proteome. The resulting PSMs for each spectrum were retained if they exceeded the 1% PSM FDR threshold in the spectrum’s original search. Library spectra with more than one distinct annotation passing the threshold were considered ambiguously identified and were removed from the library (2.5% of precursors), thus reducing MassIVE-KB to 2,122,890 precursors (Figure S3C).

To estimate the quality of representative spectrum selection, we constructed a gold standard spectral library from stable isotope labeled amino acids in cell culture (SILAC) samples (Geiger et al., 2010). Since SILAC experiments produce light and heavy versions of the same peptide (identical in retention time and fragmentation but differing only in a consistent y-ion mass shift [Mann, 2014]), this increases confidence by using concordant identification of both light and heavy MS/MS spectra. Additionally, the consistency of fragmentation between light and heavy peptide pairs can also be used to define a high standard of spectral quality, whereby we would want that (1) representative library spectra be as similar to replicate spectra from the same peptide as (2) spectra from light and heavy versions of the same peptide are similar to each other when coming from the same sample analyzed in the same MS run in the same instrument. We thus expect the MS/MS variability between light and heavy peptide pairs to be the minimum possible “ideal” variability between a library spectrum and its counterpart in the SILAC library (i.e., the spectrum for the light version of the same peptide sequence). 2,622,224 MS/MS spectra from 30 HCD LC/MS (MassIVE:MSV000079572-ProteomeXchange:PXD002098 [Deeb et al., 2012]) runs were searched and filtered with strict spectral quality requirements and consistency of fragmentation of concordantly identified co-eluting light and heavy spectral pairs (<1% PSM-level FDR) (Xu and Li, 2011) (STAR Methods: Gold Standard SILAC Library Construction). The resulting SILAC library (29,574 precursors) used to assess the quality of spectra in the library using cosine distributions to both NIST’s HCD spectral library and MassIVE-KB NIST and MassIVE-KB both exhibited high cosine similarity for corresponding precursors in the SILAC library (Figure S3A), highlighting that the MassIVE-KB reference spectrum quality matches that of the state-of-the-art libraries.

The MassIVE-KB *ab initio* construction required ~100 CPU-years of computation. Since we envisage the MassIVE-KB library growing with new public data depositions on a continuous basis, *ab initio* reconstruction with the addition of every single dataset is impractical. Rather, MassIVE-KB is augmented in an online manner (STAR Methods: Online Library Creation), that is, reusing previously computed results, reducing the computational load by over 100×, and enabling MassIVE-KB updates on a weekly instead of a yearly basis. Most importantly, our online library construction procedure guarantees that the resulting spectral library is the same regardless of the order in which the input data is used to expand the library—a key feature as otherwise the content of the spectral library could vary depending on the order in which community data are made publicly available. As such, MassIVE-KB’s library construction procedure enables computationally efficient online additions to the library while preserving the deterministic construction afforded by existing *ab initio* library construction tools. The current version of MassIVE-KB has been augmented in ten iterations, growing from 609,000 to 2.1 million precursors as the total volume of public human HCD data included in MassIVE-KB has increased from 2 TB to over 31 TB.

### Library Provenance

As spectral libraries represent the extent a proteome has been observed by MS, the provenance of the spectra in the library must be available to the community for both validation and exploration of the biological context of the original observations. Though existing spectral library resources make attempts at preserving provenance information, there is unfortunately still no way to link back to all the spectra, PSMs, searches, and software/workflows that were used to derive every entry in the spectral library.

MassIVE-KB attempts to close this gap and provide user access to a full provenance record (Figure 2; Data S1). For the entire spectral library, all LC/MS data, search jobs/parameters, and all intermediate library construction jobs are fully available to the community for detailed scrutiny. Furthermore, representative spectra, spectra considered for representative selection (top 100 candidate spectra per precursor), and the full set of PSMs used for library construction are available and point back directly to the specific LC/MS run, dataset, principal investigator (Table 1), and original search job/parameters. This transparency in the library creation process empowers the community to validate search parameters and check the knowledge in the spectral libraries against the underlying data and metadata. For example, this transparency will enable the community to discover false proteins occurring in unexpected contexts (Ezkurdia et al., 2014). Finally, we recognize that the default MassIVE-KB libraries will not cover the entire community’s needs for specialized libraries, for example, specific conditions, tissues, instruments, organisms, post-translational modifications, etc. Rather, these existing searches and libraries provide a starting point for users to customize their own reanalysis of data to create their own customized knowledge bases (STAR Methods: Documentation).

### Protein Coverage

**Figure 4.**
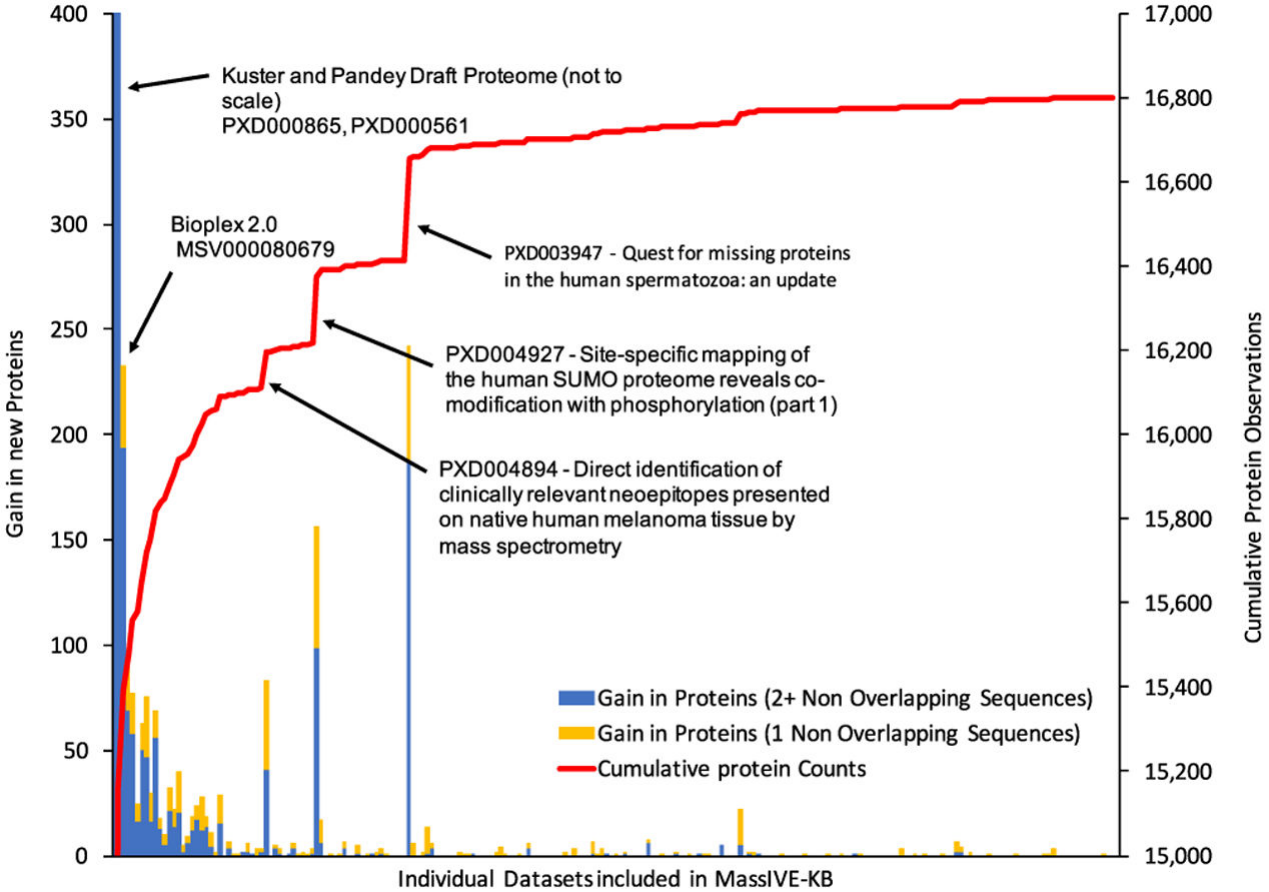
Extensive Coverage of the Human Proteome Capitalizes on Diverse Contributions from Multiple Sources as Various Datasets in the Community Contribute their Own Specific Insights into the Human Proteome The left axis shows the number of new proteins contributed by each dataset in the bottom axis (yellow bar if identified with two non-overlapping peptides, and blue if identified by only one peptide); the right axis shows the cumulative number of protein observations (starting at 15,000 for legibility; see Table S2 for details); the x axis represents all datasets included in MassIVE-KB sorted by the total proteins covered in decreasing order. Three foundational datasets (i.e., Bioplex(Huttlin et al., 2015) and the two draft proteomes [Kim et al., 2014; Wilhelm et al., 2014]) provide deep coverage of commonly observed proteins, but several smaller datasets were key in contributing unique proteins, such as the ProteomeXchange:PXD004927 small ubiquitin-like modifier (SUMO) proteome (Hendriks et al., 2017) and ProteomeXchange:PXD003947 spermatozoa (Vandenbrouck et al., 2016) proteins. Altogether, the union of community datasets (excluding synthetic peptides) supported the observation of 16,801 human proteins (83% of the human proteome).

The MassIVE-KB HCD spectral library contains evidence for 19,610 proteins (97.4%) out of the 20,129 proteins reported in SwissProt (January 2, 2017) at a 1% protein FDR (Savitski et al., 2015). Without the inclusion of synthetic peptides from ProteomeTools (Zolg et al., 2017), MassIVE-KB contains evidence for 16,801 proteins (83.4%) detected in a wide variety of public datasets (Figure 4; Table S2). This represents a gain of 48% over the NIST HCD peptide spectral library’s (July 20, 2016) 13,261 proteins (at least one unique peptide protein; undetermined protein FDR). At the precursor level, MassIVE-KB’s 2.1 million precursors represent a collection over 4-fold larger than NIST’s (Figure 3A), corresponding to 235% more unique sequences. This increases the amino acid coverage of the human proteome from 2.99 million amino acids (26.4% of the proteome) to 6.17 million amino acids (54.5% of the proteome). Furthermore, MassIVE-KB covers 196,628 exons (72.6% of all) from 19,012 genes (Figure S5) in the human genome, including 86,277 exon junctions (39.5% of exon junctions in GRCh38). MassIVE-KB additionally reports peptides that span known biologically relevant modification sites, with a gain of 57%, 105%, 47%, and 59% increase over NIST for post-translational modification sites, disulfide bond sites, binding sites, and other functional sites as annotated by UniProt Knowledgebase (UniProtKB), respectively. One such type of site, glycosylation, which serves a significant role in cellular signaling (Varki and Lowe, 2009) especially in immune cells, saw an increase of 547% over NIST’s 856 glycosylation sites covered to MassIVE-KB’s 5,540 out of a total of 17,042 reported sites by UniProtKB (Boutet et al., 2016). Most of the additional coverage leading to the contribution of these new sites was obtained from the Bioplex dataset (Huttlin et al., 2017), Lung Adenocarcinoma (ProteomeXchange:PXD002612) (Tyanova et al., 2016), human draft proteomes (ProteomeXchange:PXD000865 [Kim et al., 2014]; ProteomeXchange:PXD000561 [Wilhelm et al., 2014]), and ProteomeTools (Zolg et al., 2017) synthetic data (ProteomeXchange:PXD004732). While MassIVE-KB in many cases will not observe the biological modification on a specific peptide (since they were not searched for), MassIVE-KB does include one or more peptide sequences spanning the site. This information is meaningful in designing follow-up experiments to help identify the modified variant or for targeted MS experiments to measure the relative abundance of modified to unmodified peptides.

While some peptide sequences were unique to NIST’s HCD library (46,230 sequences), most of these were short (<10 amino acids) and represent only 1% of the proteome (Figure S3). Of the remaining 46% of the proteome not covered by MassIVE-KB, 1,035,275 amino acids (20% of the missing coverage) occur in long tryptic peptides (>40 amino acids), which are likely to be harder to recover by trypsin digestion and HCD bottom-up proteomics (Swaney et al., 2010). As such, with MassIVE-KB’s coverage of 6,167,998 amino acids and with 1,035,275 amino acids in challenging regions, we are left with 4,112,522 amino acids (36%) of the proteome remaining in trypsin-coverable regions (Figure 3D), a sizeable fraction of which were in 2,809 proteins that were only observed with ProteomeTools synthetic peptides or in 519 proteins for which there are still no representative peptides in public datasets (1,197,073 amino acids, 29%).

### Targeted Proteomics Design

Targeted proteomics experiments, for example, single reaction monitoring (SRM), aim to quantify the relative abundance of proteins in complex biological samples by measuring the abundance of constituent peptides. However, even in simple benchmark mixtures of proteins, few peptides are consistently observed across a range of samples containing the corresponding protein (Mallick et al., 2007). As such, it is important to identify protein-specific sets of consistently recurring (i.e., proteotypic) peptides, as these are the most useful for comparable quantification of proteins of interest across multiple samples. Previous analyses (Mallick et al., 2007; Kusebauch et al., 2016) have empirically predicted a set of proteotypic peptides for each protein, but the proteome coverage at the time extended to only 9,946 proteins. Using the provenance of all searches used to construct the MassIVE-KB library, we have now expanded this set to 16,801 proteins (Table S3; STAR Methods: Proteotypic Peptides).

**Figure 5.**
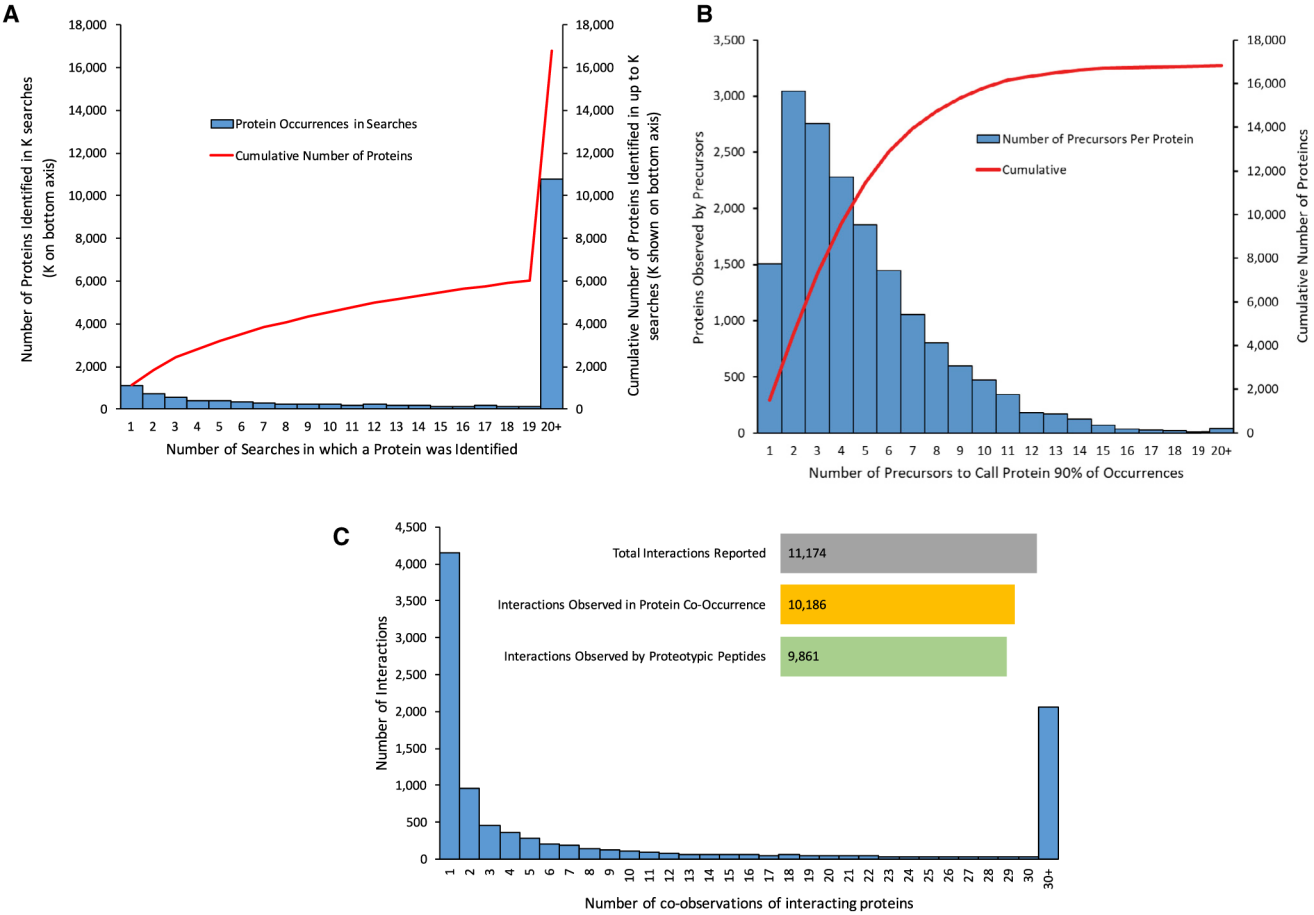
MassIVE-KB Supports Experimental Design with Frequency of Occurrences across Datasets (A) The number of times a protein was observed in all MassIVE-KB searches; this varied from a single search to over 6,000, with a median of 48 occurrences. While many proteins are commonly identified in multiple searches from various datasets, there are also thousands of proteins that are only identified in a very small number of datasets corresponding to specific experimental procedures (e.g., affinity purification) or biological conditions (e.g., tissue). (B)The number of proteotypic precursors selected such that at least one precursor is present ≥90% of the time when a protein is observed. For most proteins, four or fewer precursors are sufficient to observe a protein in 90% of its occurrences (thus reinforcing the expectation that peptide observations should be generally consistent across datasets). Upon closer investigation, the tail of the distribution further revealed that for a subset of datasets where proteotypic peptides differed drastically from the norm, the discrepancy could be partially attributed to experimental procedures for peptide enrichment (e.g., phosphorylation and SUMOylation). (C) Out of the 10,186 protein-protein interactions between primary SwissProt isoforms reported in Bioplex 1.0 that were also co-identified in the MassIVE-KB reanalysis of Bioplex data, we found that 9,861 interactions (96.8%) would continue to be detectable using only the MassIVE-KB set of proteotypic precursors.

In evaluating the consistency of precursor occurrences in samples where the corresponding protein was identified (Figure 5A), we observed a range from no recurrence (0% consistency) to always present (100% consistency). Due to this variability, the number of precursors necessary to observe each protein at least 90% of the time (Figure 5B) ranged from one to over 20, even though in most cases (58%), four or fewer precursors were sufficient to detect the corresponding proteins. This MassIVE-KB assessment of proteotypic peptides across 27,404 LC/MS runs covering 16,801 proteins is thus a valuable resource to consider when designing SRM experiments.

Furthermore, MassIVE-KB proteotypic peptides can also be used to probe protein-protein interactions in AP experiments, as the co-observation of proteins in AP experiments provides evidence that the proteins are possible interactors (Huttlin et al., 2015) (Figure 5C). Of all the 10,186 MassIVE-KB protein co-observations of reported interactions in Bioplex 1.0, we found that proteotypic peptides could detect 96.8% of them. (STAR Methods: Proteotypic Peptides).

### Detecting Unobserved Proteins

The neXtProt (Gaudet et al., 2015) resource aims to be a knowledge base that catalogs the extent of the community’s knowledge of human proteins. As such, neXtProt (February 15, 2018) claims that 17,168 proteins from SwissProt (Bairoch and Apweiler, 2000) (20,200 total) have been previously observed with protein evidence (PE1). Of these, 15,953 proteins (92.9% PE1) were observed in MassIVE-KB without synthetics at a 1% protein FDR (Savitski et al., 2015). A subset of the remaining 7.1% of proteins were manually inspected and observed to be from labeled quantification (iTRAQ) experiments and datasets (e.g., neXtProt protein NX_P98073), observed only in synthetic experiments (e.g., neXtProt protein NX_Q69383), or listed with no peptide evidence at all at neXtProt (e.g., neXtProt protein NX_E2RYF6). We envision that as more data are collected and automatically searched, these remaining proteins will become a part of MassIVE-KB.

neXtProt further labeled the remaining 3,032 proteins (PE2-PE5) to lack proteomics evidence. 430 of these 3,032 proteins were observed in the MassIVE-KB library originating from nonsynthetic data and meeting U.S. Human Proteome Organization (HUPO) guidelines (Deutsch et al., 2016) for reporting of novel proteins (i.e., with at least two non-overlapping sequences that could not map to another protein as a single amino acid variant), resulting in a protein FDR (Savitski et al., 2015) of 0.013%. The majority (380 proteins) was previously seen with transcriptomic evidence (PE2), with the remaining 13 inferred from homology (PE3), 1 uncertain (PE4), and 30 predicted (PE5) (Figure 3C). 291 newly observed proteins contained peptides that also appeared in a synthetic peptide library (STAR Methods: Synthetic Data Library Creation [ProteomeTools]) generated from ProteomeTools (Zolg et al., 2017) (Figure 3B). Nearly all MassIVE-KB precursors with corresponding entries in ProteomeTools demonstrated very high spectral similarity (4% spectra having similarity below 0.7 cosine due primarily to low-intensity synthetics), with a median cosine of 0.93 (Figure 3C). 282 of these 291 proteins (96.7%) contained peptides exhibiting >0.7 cosine to synthetic peptides, with 162 proteins containing at least two non-overlapping precursors, meeting HUPO’s extraordinary detection claims (Deutsch et al., 2016) (Figure 3B; Table S4).

## DISCUSSION

We have presented MassIVE-KB for large-scale continuous aggregation of all proteomics MS data into an open reusable knowledge base. The resulting MassIVE-KB spectral library delivers a provenance-controlled record of community-scale proteomics knowledge supported by global proteomics data and is readily usable to support future discoveries in both shotgun and targeted proteomics experiments. As new studies and data are deposited in MassIVE and other ProteomeXchange repositories, they will be automatically searched and appended to the existing spectral library in an online and error-controlled manner, with full provenance records open to community inspection and revision.

The MassIVE-KB spectral library is freely available to be redistributed and reused in several ways (Figure 2): (1) downloaded as an annotated MGF for use with ProHits (Liu et al., 2010) and for further computational reanalyses; (2) downloaded as an sptxt library file for use with SpectraST (Lam et al., 2007) in the Trans-Proteomics Pipeline (Deutsch et al., 2010) and Skyline (MacLean et al., 2010); and (3) used in CCMS’s computational workflows engine for online library searching of any public or private proteomics MS data, including MSPLIT (Wang et al., 2010) (data-dependent acquisition [DDA] spectral library search), MSPLIT-DIA (Wang et al., 2015) (data-independent acquisition spectral library search), and Maestro (spectral networks analysis). To evaluate the potential gains of MassIVE-KB spectral library searches, we observed that searching a recent HEK293 dataset (Chick etal., 2015) containing 1.1 million MS/MS spectra with MSPLIT showed that MassIVE-KB identified 64% more precursors than the exact same search using NIST’s HCD library instead of MassIVE-KB (Figure S4; STAR Methods: Decoy Inclusion in Spectral Library, Spectral Library Search).

While the MassIVE-KB spectral library is a general-purpose resource for the proteomics community, we also expect that there will be a need to construct spectral libraries for other species and for more targeted (e.g., neuropeptides) or specialized (e.g., tandem mass tag [TMT]-labeled peptides) purposes. As such, MassIVE-KB also makes its workflows to search and construct spectral libraries freely available to the community for user-driven construction of spectral libraries. As an illustration of the potential of this approach, we also constructed a MassIVE-KB spectral library containing only synthetic peptides from the recent ProteomeTools synthesis of hundreds of thousands of peptide sequences. Leveraging the provenance of the MassIVE-KB spectral libraries, the community can use the existing search and library construction workflows as a starting template to create their own distilled and reusable library resources. Altogether, MassIVE-KB’s structured data, spectral libraries, algorithms, and provenance-tracked online process for continuous aggregation of proteomics discoveries in an error-controlled manner, all come together to deliver a communityscale platform, further supporting the translation of proteomics big data into open and reusable proteomics knowledge.

## STAR★METHODS

### CONTACT FOR REAGENT AND RESOURCE SHARING

Further information and requests for resources and reagents should be directed to and will be fulfilled by the Lead Contact, Nuno Bandeira (bandeira@ucsd.edu).

### METHOD DETAILS

#### Data Origin and Preparation

The initial construction of the Massive-KB spectral library used 31TB of human HCD data from 227 public datasets. All data is available at MassIVE (Table S1); the Bioplex dataset was obtained directly from the Bioplex project and, to support the provenance records of the search results, the reanalyzed files are now also made available as a new dataset (MSV000080679). All raw files were converted using ProteoWizards’ msconvert (Kessner et al., 2008), configured to output centroided 32-bit uncompressed mzXML or mzML (Deutsch, 2008) files (online workflow available at MassIVE); all resulting files are made available at MassIVE as a part of the respective dataset (subdirectory “ccms_peak” in each dataset).

#### Single Pass Search

MSGF+(Kim and Pevzner, 2014) was used to search tandem mass spectra against the UniProt human reference proteome database (May 23, 2016) containing 70,625 proteins. Allowed variable modifications were oxidation on methionine (M+15.995), N-term acetylation (+42.011), N-term Carbamylation (+43.006), Pyro-glu on glutamine (−17.027Q), and deamidation on aspargine (N+0.984) and glutamine (Q+0.984). Carbamidomethylation was also searched as a fixed modification on cysteine (C+57.021). MSGF+ database search was configured to allow one ^13^C precursor mass isotope, at most one non-tryptic termini and 10 ppm precursor mass tolerance. Unless otherwise noted, each individual search was filtered to 1% PSM-level FDR.

#### ProteomeTools Synthetic Searching

Synthetic peptide spectra from ProteomeTools(Zolg et al., 2017) were pooled into 365 samples of 1,000 peptides; each pool of 1,000 peptides was acquired multiple times to cover different fragmentation modes and collision energies. Each LC/MS run with scheduled HCD acquisition was separately searched by MSGF+ against a small target database consisting of the sequences of the peptides in the corresponding pool and a MSGF+-generated small decoy database with the reversed version of the target database sequences(Kim and Pevzner, 2014). Since this decoy database is too small to properly estimate false discovery rates, we also added 10 randomly-selected ProteomeTools LC/MS runs to each search to provide a background of knowingly-false matches: any spectrum matched from these runs is known to be a false match because the sequence of its peptide is not supposed to be in the target database. A decoy PSM was thus defined as: 1) a spectrum from the main pool matched to the decoy database or 2) a spectrum from the background pools matched to either the target or decoy database. Peptides that were synthesized in each background peptide pool and also appear in the target database, were excluded from possibly being counted as a decoy PSM. The remaining PSMs from each ProteomeTools search (including decoys from background LC/MS runs) were sorted by increasing database p-value and filtered to 0% empirical PSM-level FDR by peptide length (i.e., the threshold was set to exclude all decoy matches).

#### Dynamic Search Space Adjustment Search

To improve the sensitivity of searches and take advantage of the experimental metadata, our MSGF+ search workflows dynamically adjusted the effective size of the search space for different types of searches. This adjustment uses the MSGF+ concept of a database p-value(Gupta et al., 2011) (STAR Methods: Database p-Value Calculation) accounting for both the quality of the peptide-spectrum-match (which is a feature of only a spectrum and the sequence assigned to the spectrum) and the size of the search space (which depends on the biological and experimental context of the observation of a spectrum). As a result, the exact same peptide assigned to the exact same spectrum can have a database p-value that is three orders of magnitude smaller (i.e., better, Figure S1C) if the PSM is derived from a synthetic peptide instead of from a large-scale proteomics experiment with millions of spectra from hundreds of thousands of distinct peptides.

In difference from MSV000080544 (ProteomeTools synthetic peptides), each proteome dataset was searched against the reference human proteome as a single unit, with the exception of the draft proteomes (MSV000079526 and MSV000079514, with one search per multi-fraction mass spectrometry experiment) and Bioplex (MSV000080679, one search per bait). Since the affinity purification (AP) runs in the Bioplex dataset also have less proteins per sample than full cell lysates, database p-values were also adjusted to reflect the correspondingly smaller sequence search space. As such, a peptide p, which can be either a target or decoy peptide, is considered identified in the reduced AP search space if its corresponding protein P is also identified in the same search by at least one other peptide p’ from the same protein P (at 1% peptide-level FDR). To properly model the score adjustment of p-values of false matches to target, the score of a decoy peptide p is also adjusted if at least one other peptide p’ comes from either the same decoy protein P (models false/false matches to target) or from the corresponding target protein from which the decoy protein was generated (models true/false matches to target). P-values for peptides matched to proteins for which there was only one identified peptide were not readjusted to the reduced search space and retained their original database p-values. Results are then filtered to 1% PSM level FDR with database p-value as the primary score ranking.

#### Spectrum Extraction/Filtering

All PSMs passing FDR in each search were considered eligible for addition to the spectral library. For each PSM, the corresponding MS/MS spectrum was extracted from the appropriate file and fragment peak intensities were rescaled to Euclidean norm of 1.0. Fragment peaks were annotated using the standard ion labels(Roepstorff and Fohlman, 1984): “b”, “b-isotope”, “y”, “y-isotope”, “b-H2O”, “b-NH3”, “y-H2O”, “y-NH3”, and “a” with fragment charges up to and including the precursor charge and with a mass tolerance of 0.1 Da; these were then used to discard all spectra with less than 40% fragment intensity explained by labeled peaks. Further, any MS/MS spectrum whose precursor mass deviated from the theoretical mass by more than 50 ppm were filtered out, including any ^13^C isotopes.

#### MassIVE-KB Spectral Library Precursor Selection

For each precursor (i.e., combination of peptide sequence and charge), the top 100 spectra with the best (lowest) database p-values were retained (with a maximum of 20 per dataset to reduce bias) for further consideration. Selected precursors were binned by peptide sequence length, ranked by increasing database p-value in each bin and filtered to 1% local FDR using a sliding window of 500 precursors (i.e., the threshold T is set to the highest database p-value where there are <1% decoys in the window around the precursor with database p-value T). This resulted in global precursor-level FDR of 0.1%.

Precursors differing only by the site localization of modifications were removed and only the highest-scoring precursor was retained. Remaining precursors were then mapped to the SwissProt Human Proteome (Jan 2, 2017) and protein level FDR was estimated by TDA(Savitski et al., 2015) using only non-shared precursors whose sequences mapped to only one protein. Each protein P was scored by the sum of the −log_10_ of each precursor’s database p-value that maps to P, which is equivalent to scoring P by 1 minus the product of the database p-values of all precursors mapped to P (i.e., the score for protein P is inversely proportional to the probability that all the evidence for P was observed by random chance). Proteins were then filtered to 1% protein-level FDR(Savitski et al., 2015) and precursors were retained if and only if their peptide sequences mapped to at least one protein in the filtered set.

To select a representative spectrum for each retained precursor, we calculated the cosine similarity(Stein and Scott, 1994; Lam et al., 2007,^2008^; Wang et al., 2010; Yang et al., 2014; Shao and Lam, 2017) between all pairs of spectra in the top 100 per precursor and set the representative library spectrum to the experimental spectrum with the highest average similarity to all other spectra from the same precursor. The performance of this procedure used to select reference library spectra was evaluated using a gold standard spectral library constructed from SILAC experiments (STAR Methods: Gold Standard SILAC Library Construction) and showed to yield representative spectra that are comparable in quality to those in the state-of-the-art NIST spectral library (Figure S3).

#### Filtering Ambiguous Library Spectra

To remove spectra with uncertain peptide identifications (i.e., more than one peptide with a significant match to the spectrum), representative library spectra were searched again against the UniProt human reference proteome database (May 23, 2016) by MSGF+ using the settings described above. The top 20 identification candidates were considered for each library spectrum and we then retained all PSMs with a database p-value under the 1% PSM-level FDR threshold in the library spectrum’s original search (since search-level FDR thresholds are less strict than the library-level FDR threshold, this yields a more conservative assessment of ambiguity by potentially retaining more PSMs per spectrum). As before, PSMs differing only by site localization of modifications were removed and only the best-scoring PSM per variant was retained. Finally, library spectra left with two or more PSMs passing the FDR threshold were labeled as ambiguous and were removed from the library.

#### Online Spectral Library Augmentation

As new datasets become available, they are converted to open formats, searched with the same database search workflows and identified spectra are extracted as described above. Newly-available annotated spectra are then merged with the existing top 100 PSMs per precursor (possibly displacing older spectra) and the full set is again filtered to 1% length-dependent precursor-level local FDR to determine a new database p-value threshold. We then repeat the steps described above for library-level protein FDR, selection of representative spectra and removal of ambiguous spectra. Most importantly, we note that i) this process does not require any re-searching of (the potentially very large volumes of) data used to construct the previous version of the library and ii) guarantees that the resulting library is consistently the same regardless of the order in which new datasets are made publicly available and added to the library (STAR Methods: Online Library Creation).

#### Novel Protein Calling

Precursors identified to peptide sequences of length >=9 were mapped to the SwissProt (Jan 2, 2017) human proteome (20,129 reviewed proteins) and filtered to 1% protein-level FDR as described above. In accordance with HUPO guidelines(Deutsch et al., 2016) for the identification of novel proteins, peptides mapping to known proteins (Protein Existence level 1(UniProt Protein existence, December 2017), or PE1) with a single amino acid variation were removed from consideration. Novel proteins (PE2 to PE5) were then retained if they were matched by at least two non-overlapping peptides.

#### Database p Value Calculation

The database p-value (Gupta et al., 2011) captures the probability of at least one high scoring false match to a given spectrum in a database of size N. Lower database p-values indicate better matches.

The equation to calculate the database p-value is as follows:

$$1-{\left(1-P(S|T)\right)}^{N},$$

where P(S∣T) represents the spectral probability (Kim et al., 2008), that is the probability of a random peptide match to spectrum S with a score higher than T. This value captures how well a peptide matches a spectrum S, with smaller values being a better explanation. 1 − *P*(*S∣T*) represents the probability of a random match with worse (higher) score than T. N represents the size of the database as the total number of candidates that are eligible to match against the spectrum S. (1 − *P*(*S∣T*))^*N*^ is the probability that all random database matches are of bad (higher) score. Then the final expression 1 − (1 − *P*(*S∣T*))*^N^* is the probability that *at least* one random database peptide match to spectrum S with a score better (lower) than T. Since lower database p-values indicate higher confidence, we can aim to lower the database p-value in several ways: decreasing spectral probability *P*(*S∣T*) and decreasing database size (N). To decrease spectral probability, one can acquire higher quality spectra or find a peptide sequence that better explains the spectrum. To decrease the database size in samples where the true set of peptides and proteins that occur is unknown, we discover the appropriate database (see STAR Methods: Dynamic Search Space Adjustment Search). When the database is known, e.g. synthetic peptide pools, we specifically constrain the database size and calculate the database p-value appropriately (STAR Methods: Synthetic Data Library Creation [ProteomeTools]).

#### Dynamic Search Space Adjustment for Proteome Limited Data

The dynamic adjustment of search space (see STAR Methods: Dynamic Search Space Adjustment Search) for affinity purification mass spectrometry searches results in a reduction of the search space from 70,625 proteins in the UniProt Human Reference database (May 23, 2016) down to an average of ~1,500 proteins (Figure S1D). PSMs that match to these proteins have their scores adjusted because of the increased confidence in the reduced search space while all other PSMs scores remain unchanged. Because of the requirement that two distinct sequences must match to a protein for the protein to be included in the second pass search set, a single PSM cannot result in adjustment of its own score (rather a second distinct PSM of a different sequence is required). To properly model false discovery rates using the target/decoy approach (TDA; Elias and Gygi, 2007) within the set of peptides with adjusted scores, we allow a protein P to be selected to the second pass search if there is a total of 2+ peptides matched to either the target sequence P or the corresponding decoy sequence reverse(P). While selecting proteins where two peptides map to the same sequence allows TDA to properly model false/false matches of peptide pairs to target (by selection of false/false matches to decoy sequences), this would not properly consider true/false cases where one peptide is a true match to target but the second peptide is a false match to target. As such, our approach of adjusting scores based on whether the two peptide matches come from either the target protein P or its corresponding decoy reverse(P) extends TDA to model true/false cases by allowing the second (false) peptide to be a decoy match (thereby modeling cases when the second peptide is a false target match).

By appropriately adjusting the search space for the samples from Bioplex, the MassIVE-KB spectral library increased by 172K precursors (8% of the MassIVE-KB library and 35% of Bioplex’s unique precursors).

#### Global Error Rate Controls for Spectral Library

In response to the accumulation of errors in repository wide aggregation of identification results, we globally controlled error rates at the precursor and protein levels. Per search, we filtered to 1% PSM level FDR. However, if we naively aggregated all PSMs, the precursor FDR would grow to 28%. Thus, we further filter precursors to a 1% local precursor FDR by length. Each precursor’s score is the best score of any PSM from that precursor. All precursors are grouped by length and then sorted by decreasing score (-log(database p-value)). The empirical local FDR (as determined by TDA) (Kall et al., 2008) of a precursor is the ratio of decoys to targets of a precursor’s immediate neighboring 500 (higher scoring) precursors.

Following precursor FDR, we calculated picked protein FDR (Savitski et al., 2016) and retained only precursors that mapped to SwissProt proteins falling below 1% protein-level FDR. A protein’s score is the sum of −log(database p-value) for all uniquely mapped precursors (conceptually equivalent to the product of database p-values for all uniquely mapped precursors). By searching UniProt Reference initially and only considering precursors mapping to SwissProt proteins, we enable a more conservative FDR calculation, as was recently shown in Sticker et al (2017; PMID: 28661493).

All candidate spectra in consideration for being the representative spectra in MassIVE-KB (top 100 scoring spectra per precursor) uniformly had a TDA-estimated FDR of 0% in the original searches (we note that this is an estimation artifact due to the lack of decoy matches at the TDA-selected score thresholds; the real FDR is expected to be very small but is likely greater than zero). Thus, if we had chosen a TDA-determined 0% PSM level FDR on a per search basis the resulting library would not have changed. However, we opted to include decoys from the initial searches because that enabled us to empirically estimate global library FDRs.

#### Gold Standard SILAC Library Construction

Since it is generally not possible to define the ‘perfect’ spectrum for a given peptide precursor, we constructed a gold standard spectral library where the consistency of MS/MS fragmentation patterns between light and heavy versions of the same precursors was used to define the ideal similarity between MS/MS spectra. Intuitively, a reference library spectrum for peptide P is considered high quality if it matches to the SILAC library spectrum L for P with a score comparable to the match between L and the SILAC-labeled heavy version of the same peptide P. SILAC datasets were searched using the same MSGF+ settings described in Online Methods and with the additional SILAC variable modifications of +8.0142 on Lysine and +10.0083 on Arginine. Results were filtered to 1% PSM level FDR and concordant spectral pairs of light and heavy versions of the same peptide precursor were determined using the following criteria:

1. MS/MS spectra must be assigned to the exact same peptide sequence except for the SILAC modification
2. Aligned cosine score (wang et al., 2016) greater than 0.6
  - Peaks between light and heavy spectra are aligned at the same mass or at a mass delta. Delta is equal to the parent mass difference divided by z, where z = {1,2 ... precursor charge}. The alignment between peaks of light and heavy spectra that maximizes the cosine score is chosen.
3. Explained Intensity greater than 0.5 in both spectra
4. Retention time difference of at most one minute

Light version of concordant light/heavy pairs is considered for inclusion in the SILAC library. The best (lowest) database p-value scoring spectrum for each precursor is selected to be the representative in the library.

#### Online Library Creation

The MassIVE-KB spectral library can be augmented in an incremental manner. When new datasets become available, they are automatically searched, PSMs extracted, and augmented to the existing library. We aim to minimize the amount of redundant computation during the addition of new data. The online addition process is performed in such a manner that is agnostic to the order in which datasets are added to MassIVE-KB, i.e. the resulting MassIVE-KB is identical even if the order of addition is permuted. This order agnosticism is maintained for both the determination of precursors for inclusion in MassIVE-KB and the candidate representative spectra for consideration.

A full list of precursors is maintained (unfiltered and including decoys) with the best scoring PSM for that precursor recorded. The precursor FDR threshold is recalculated whenever new data is added. The top 100 candidate representative spectra are maintained regardless of whether the precursor exceeds the FDR threshold to be included in MassIVE-KB. Only at the time of representative selection are precursors excluded and low scoring PSMs dropped from consideration. Maintaining both of these data structures ensures order invariance with respect to when new data is added to the library. We have empirically tested order permutation of dataset additions to the MassIVE-KB with identical resulting spectral libraries.

This stability contrasts with SpectraST’s construction process (Lam et al., 2007), where precursors can be lost if new additions become too similar to existing library entries regardless of the confidence of the identifications.

#### Proteotypic Peptides

Dataset files were grouped as a unit and searched as a unit. The human draft proteomes were broken up based on experimental data, i.e. by tissue and experiment unit. Each unit of data was searched by MSGF+ using the same search parameters for library construction. The set of PSMs for each dataset was filtered by the minimum score cutoff for inclusion in the MassIVE-KB spectral library (i.e. minimum score threshold by peptide length). Unique peptides in this remaining set of PSMs determined the set of proteins called in each search.

The set of searches in which a protein occurred was then processed as follows. We select one proteotypic precursor at a time for each protein, choosing the precursor which covers the greatest number searches not already covered by the existing proteotypic set of precursors. The process stops when 90% of a protein’s searches are covered by the proteotypic set of precursors or the maximum of twenty precursors is reached.

To determine the set of protein-protein interactions observed in the data, we searched each AP-MS bait from the Bioplex project separately. Again, all PSMs are filtered by minimum score thresholds for inclusion into MassIVE-KB. Proteins are called by unique peptides in each search. A possible interaction is detected if two proteins are co-observed in the same pulldown.

Out of 11,174 interactions reported by Bioplex 1.0 between primary isoforms of proteins in SwissProt, 990 protein-protein interactions were not observed in our analysis of the Bioplex data. 274 were because of the absence of the protein in MassIVE-KB. Another 253 interactions included proteins that contained fewer than five precursors and thus might have been possible to miss these identifications in the data. The remaining 463 interactions require further investigation as many of the proteins contained a high number of observable precursors, but yet were missed in the Bioplex searches. We hypothesize that it could possibly be attributed to the stricter global precursors FDR requirements for MassIVE-KB compared to the original analysis or that shared peptides might have been used to infer the presence of the interactions.

#### Synthetic Data Library Creation (ProteomeTools)

To help validate the new proteins observed in the MassIVE-KB library before the inclusion of synthetic data, we created a spectral library from only the ProteomeTools MS/MS data. As described in STAR Methods, all LC/MS runs that contained HCD spectra (730 LC/MS runs) were search (see STAR Methods: ProteomeTools Synthetic Searching). Results were filtered to 0% search FDR and the standard pipeline for library creation was used (Figure 2). This spectral library contained 268,619 precursors and is available to browse online. While ProteomeTools reported a library size of 268,339 sequences in their manuscript, no library was provided. However, the in house ProteomeTools library only held 204,780 (76%) sequences. We anecdotally observed that many of the missing sequences fall below our FDR thresholds with very poor scores, indicating that either the synthesis was unsuccessful for a subset of peptides or were detected in other ionization modes and missed in HCD.

Further, there only appears to be a very slight bias of missing peptides skewed in favor of peptides from PE1 proteins. 22.1% of peptides that should have been from PE1 proteins were missed in CCMS’s ProteomeTools Library, whereas 29.8%, 30.6%, 28.9%, and 28.9% were missing from PE2, PE3, PE4, and PE5 respectively.

#### Decoy Inclusion in Spectral Library

The source of errors in spectral library search identifications arise from erroneous spectral matches and incorrect identifications of library spectra. The former is captured by spectral decoys (Lam et al., 2010) in library search, but the latter is generally ignored to exist. To address this, in MassIVE-KB the database search decoys are carried throughout to the library search process to allow for controlling this type of error. Thus, this introduces another class of decoys for library search to consider (incorrect identifications of library spectra). We have implemented this awareness into an MSPLIT (Wang et al., 2010) workflow that is available to use online at CCMS. While the MassIVE-KB library contained only 1,910 (0.1% of library) decoy precursors, these decoys constituted 6% of all decoy matches is the search of 1.1M MS/MS spectra from a HEK293 HCD dataset (STAR Methods Spectral Library Search), an over-representation of over 60x.

#### Spectral Library Search

The MassIVE-KB spectral library is distributed both as an MGF and sptxt format for use in various tools. In its MGF format, it is directly compatible with the MSPLIT (Wang et al., 2010) spectral library search workflow available at CCMS. A HEK293 dataset (Chick et al., 2015) containing 1.1M MS/MS spectra was searched with both the MassIVE-KB library (Search task) and NIST’s HCD library (Search task). Though this dataset was used in creating the MassIVE-KB spectral library, any representative spectra from this dataset was removed from MassIVE-KB for these searches. MassIVE-KB increased precursors (at 1% precursor FDR) identified from 77,472 to 127,419 (Figure S4), a 64% increase.

### DATA AND SOFTWARE AVAILABILITY

#### MassIVE-KB Availability

The latest versions of MassIVE-KB are available athttp://massive.ucsd.edu/ProteoSAFe/static/massive-kb-libraries.jsp. A list of all releases of the MassIVE-KB HCD library and other supporting data are encapsulated in the MassIVE datasetMSV000081142.

#### Documentation

Online documentation for users to browse MassIVE-KB and to create their own libraries is available athttp://proteomics.ucsd.edu/massive-kb-repository-scale-spectral-libraries/

#### Source Code

Source code and Proteosafe workflows can be found on Github athttps://github.com/CCMS-UCSD and at https://github.com/sangtaekim/msgfplus.

## Supplementary Material

1

2

3

4

5

6

## Figures and Tables

**Table 1.** Top Ten Groups Contributing to the Most Proteomics Mass Spectrometry Data to MassIVE-KB

Proteomics Group	Contributions to Representative Library Spectra	Contributes to Candidate Library Spectra
Steven Gygi	660,801 (30.4%)	5,135,165 (17.0%)
Bernhard Kuster	252,229 (11.6%)	4,570,424 (15.1%)
Matthias Mann	242,048(11.1%)	3,556,504 (11.8%)
Akhilesh Pandey	172,966 (7.9%)	2,356,169 (7.8%)
Christopher Gerner	68,389 (3.1%)	1,277,260(4.2%)
Wisniewski Jacek R.	51,844 (2.4%)	541,647 (1.8%)
Michael Nielsen	42,929(1.9%)	645,759(2.1%)
None Listed	39,571 (1.8%)	499,093 (1.7%)
Christophe Bruley	37,188(1.7%)	303,185 (1.0%)
Tamar Geiger	36,163(1.7%)	1,046,788 (3.4%)

While these ten groups contributed most of the representative spectra in MassIVE-KB, another 110 proteomics groups collectively contributed the remaining 32.1% of all MassIVE-KB candidate spectra. This large tail strongly emphasizes the significance of each community member’s contributions and reinforces the MassIVE-KB approach that a comprehensive knowledge base should be compiled from the collective contributions of the entire proteomics community.

**Table:** KEY RESOURCES TABLE

REAGENT or RESOURCE	SOURCE	IDENTIFIER
Deposited Data		
Raw and Processed Data is available at MassIVE, a ProteomeXchange repository at http://massive.ucsd.edu	Various	See Table S1
Software and Algorithms		
MassIVE-KB Construction Software and Workflow	This Manuscript	https://github.com/CCMS-UCSD/MassIVEKBWorkflows

## Highlights

- Reprocessed 31 TB of human proteomics data
- MassIVE-KB spectral library including 2.1 million precursors (>4-fold increase)
- 55% of all human proteome amino acids are covered (2-fold increase)
- 430 new proteins observed with previously missing proteomics evidence
