# Supplementary material for: Assembling the Community-Scale Discoverable Human Proteome
Source: Cell Syst. Author manuscript; Available in PMC 2019 Oct 24. (PMC6279426; doi:10.1016/j.cels.2018.08.004)
Supplement: 1 [file NIHMS1504990-supplement-1.pdf]

Cell Systems, Volume 7

Supplemental Information

Assembling the Community-Scale

Discoverable Human Proteome

Mingxun Wang, Jian Wang, Jeremy Carver, Benjamin S. Pullman, Seong Won Cha, and Nuno Bandeira

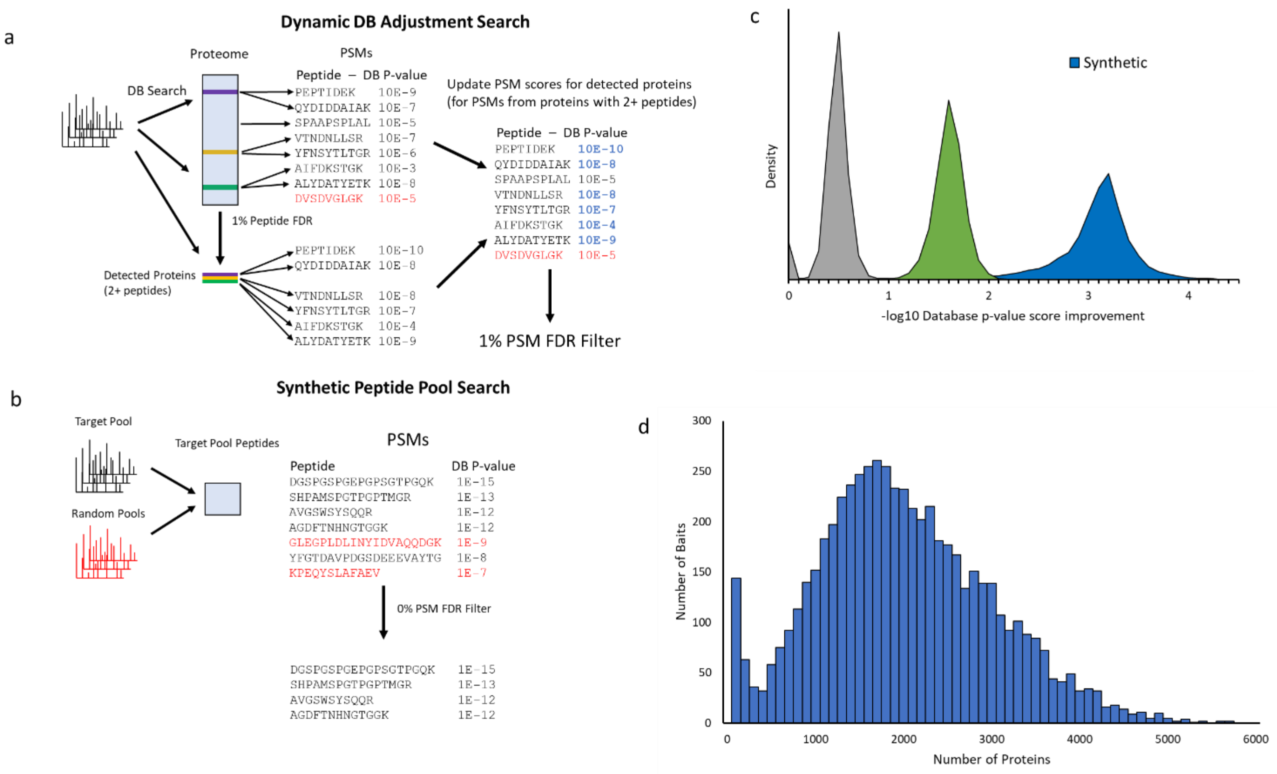

Supplementary Figure 1 - Technical Search Overview and search space modeling, Related to STAR Methods. (a) Illustration of search strategy for protein constrained dataset (e.g. affinit y purification). Spectra are first searched against the full proteome and filtered to 1% peptide level FDR. Proteins with two or more peptides are selected and PSMs are re-scored against this reduced protein set to update database p-values for spectra identified to these proteins. Results are filtered to 1% PSM level FDR to retain enough decoys for estimation of library-level FDR. (b) Each target pool of synthetic peptides was searched along with 10 randomly-selected synthetic peptide runs against the appropriate peptide database containing the target synthetic sequences. All identifications from the target peptide pool data are counted as targets and identifications to the decoy database or from the random peptide pool data are counted as decoys. All PSMs are ranked by their database p-value scores and all PSMs with a q-value greater than 0 are removed. We chose to not include decoy spectra in the library as they will always be removed subsequently in the ambiguity step as the correct peptide sequence match will always supersede the decoy annotation. (c) The appropriate search space for a mass spectrometry dataset can vary widely based on experimental procedures, ranging from searching nearly the full proteome for cell lysates down to only tens or hundreds of peptides for synthetic peptide pools. By appropriately modeling this search space for cell lysate, affinity purification, and synthetic peptide pools, database p-value scores improved by ~0.3, ~1.6, and ~3.2 orders of magnitude respectively. (d) Each bait in the Bioplex AP-MS dataset was searched separately. Proteins were filtered per search to 1% protein FDR. The distribution of proteins identified by 2+ peptides in refined search spaces is centered on approximately 1,500 proteins, down from 70,625 proteins in the Uniprot Reference Proteome (**Methods: Dynamic Search Space Adjustment Search**).

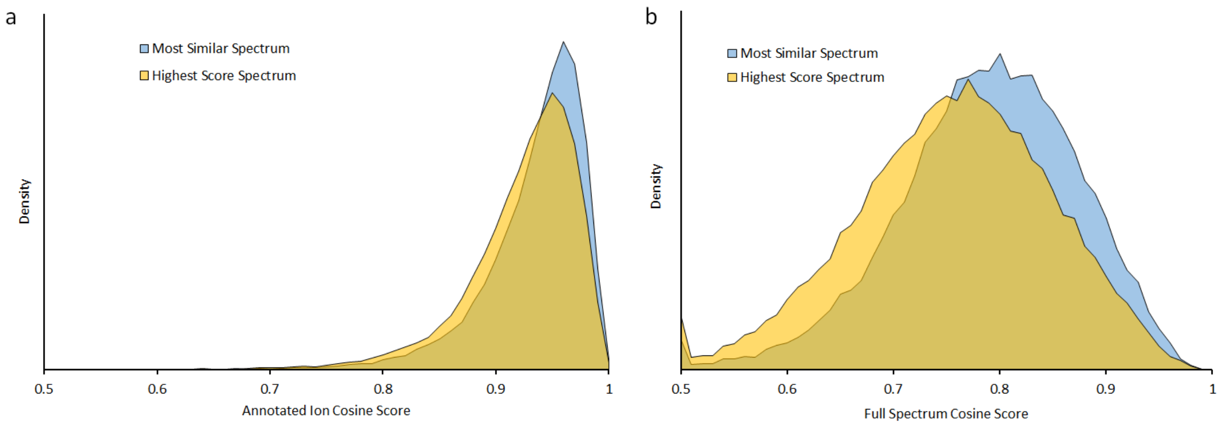

Supplementary Figure 2 – Comparison of methods for selecting spectral library representatives, Related to STAR Methods. Setting spectral library representatives to the spectrum with the highest average similarity to all replicate PSMs from the same precursor yields a better cosine similarity to the gold standard SILAC library than simply setting the spectral library representative to the highest scoring (i.e., lowest database p-value) replicate spectrum.

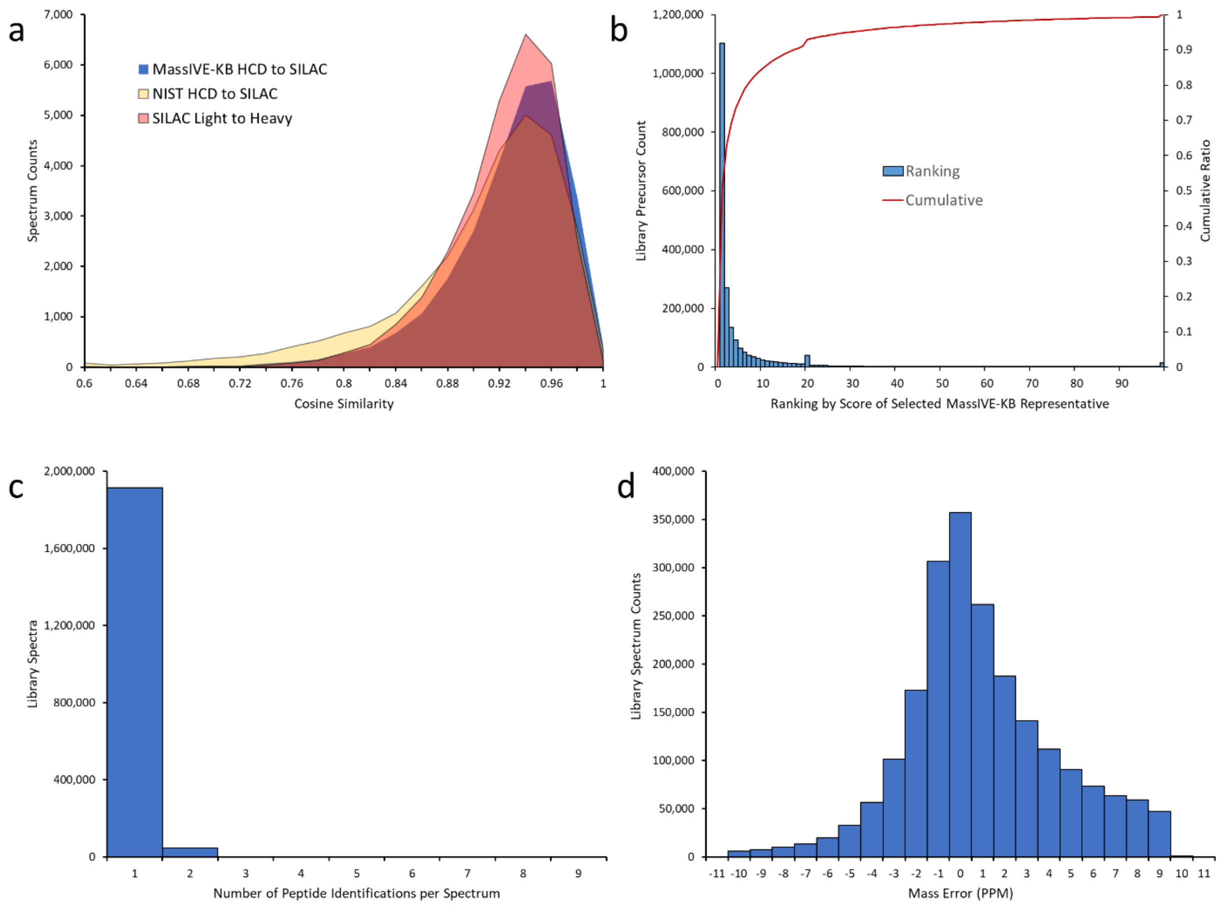

Supplementary Figure 3 - MassIVE-KB Library Quality, Related to STAR Methods. (a) Spectral similarity of annotated ions in representative spectra in the NIST and MassIVE-KB spectral libraries compared to the SILAC gold standard library. Shown in red is the distribution of cosine scores between light and heavy SILAC MS/MS spectra representing the best similarity that can be expected from replicates. (b) Per-precursor rank of the spectra selected as spectral library representatives for all entries in MassIVE-KB; in >90% of cases the selected representative spectrum was in the top 20 spectra per precursor. (c) The number of peptide identifications passing FDR for each candidate library spectrum. Only 2.5% of library spectra were found to be ambiguous (i.e., two or more PSMs passing the per-search FDR threshold) and were removed from the MassIVE-KB spectral library. (d) Precursor PPM error of library spectra.

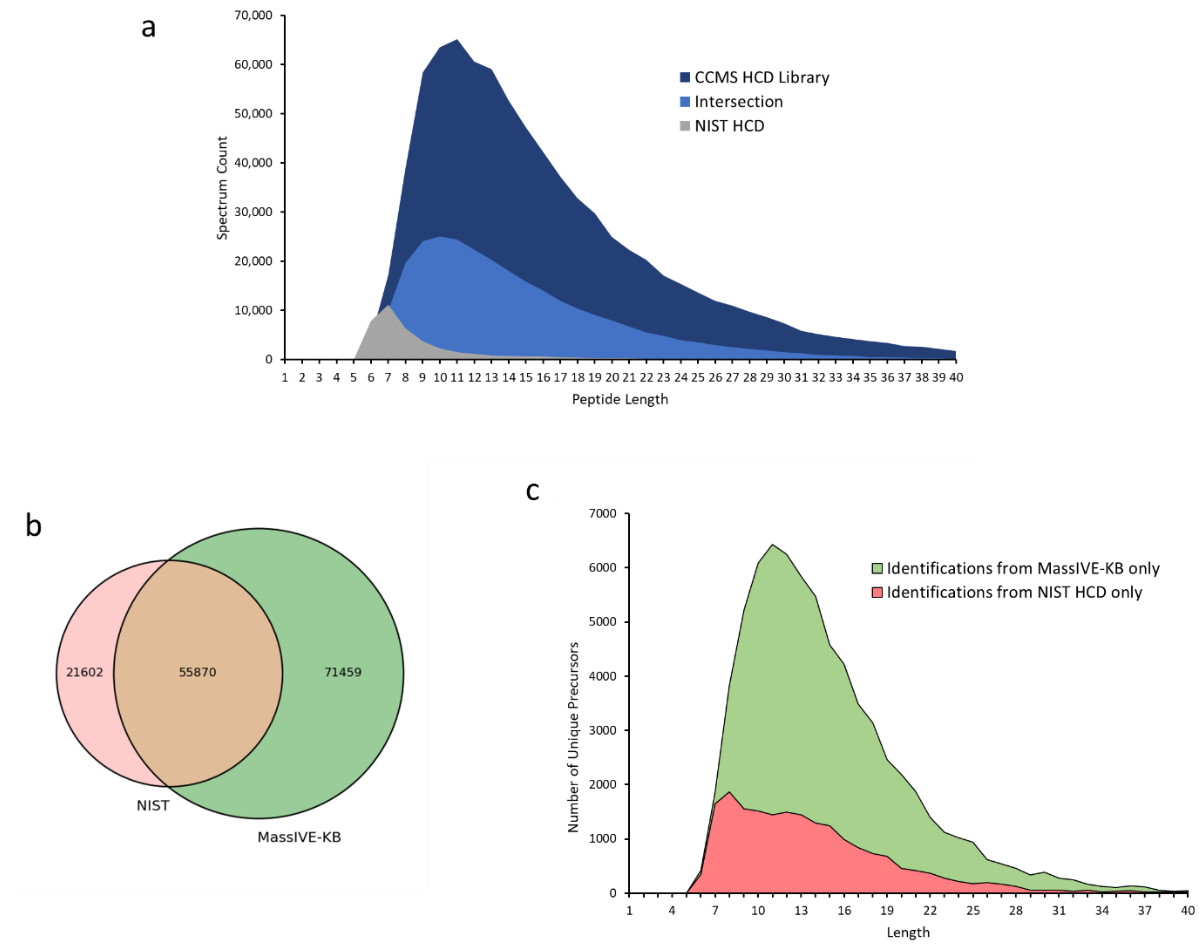

Supplementary Figure 4 - MassIVE-KB and NIST spectral libraries comparison, Related to Figure 3. (a) While NIST contains 50K unique sequences not contained in the MassIVE-KB libraries, the majority (62%) are from short peptides (<9 amino acids). In contrast, the gains in sequences in MassIVE-KB library over NIST are predominantly longer sequences that are more commonly observable and identifiable in mass spectrometry. (b) Using the MassIVE-KB spectral library to search a HEK293 Q-Exactive dataset (PXD001468) increases the number of precursors identified by 64% (MSPLIT MassIVE-KB search) over NIST's HCD library (MSPLIT NIST HCD search); at 1% precursor-level FDR, MassIVE-KB identified 127,419 unique precursors and NIST's HCD library identified 77,029 unique precursors. (c) As with the libraries as a whole (a), the precursors uniquely identified by the NIST HCD library trended toward shorter peptides that were missing from the MassIVE-KB library.

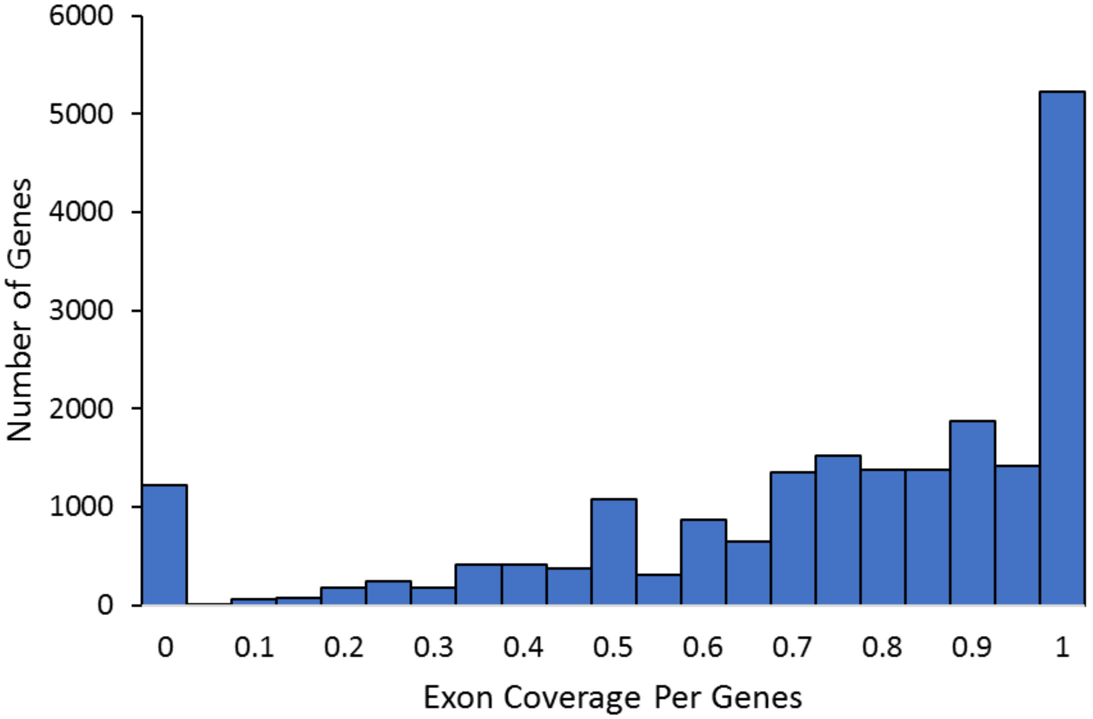

Supplementary Figure 5 – Exon coverage per gene, Related to Figure 3. MassIVE-KB uniquely (with peptides mapping to a single location in exons) covers 196,628 exons (72.6%) with at least 9 nucleotides per exon from ENSEMBLE transcripts[1] (downloaded July 5, 2017) and the Genome Reference Consortium Human genome build 38 (GRCh38). This represents 19,012 genes (93.9%) each with a varying percentage of exons covered per gene.

1. Flicek, P. *et al.* Ensembl 2012. *Nucleic Acids Res.* **40,** (2012).
