# Supplementary material for: Assembling the Community-Scale Discoverable Human Proteome
Source: Cell Syst. Author manuscript; Available in PMC 2019 Oct 24. (PMC6279426; doi:10.1016/j.cels.2018.08.004)
Supplement: 6 [file NIHMS1504990-supplement-6.pdf]

Data S1

Note 1. All Protein Observations in MassIVE-KB

The latest versions of the MassIVE-KB are available at http://massive.ucsd.edu/ProteoSAFe/static/massive-kb-libraries.jsp.

All provenance information is accessible for each MassIVE-KB in this link:

Download	Library Name	Description	Precursors	Peptides	Proteins
MGF | sptxt	Human HCD Spectral Library	This is the Human HCD spectral library. Containing over 2.1M precursors and drawing on over 30TB of MS/MS proteomics data.	2,122,892	1,088,645	19,610

To view all proteins in MassIVE-KB, click on this link:

Proteins Results
[All Proteins]

All proteins in SwissProt are listed in this view along with statistics of observation in MassIVE-KB. Specifically, to view all PE2+ proteins by neXtProt, filter the "neXtProt PE" minimum to 2. To determine the observed proteins in MassIVE-KB according to HUPO criteria, filter the "HUPO Non-Overlapping Peptides" to minimum 2.

To observe all HUPO compliant peptides that confirm the existence of each protein, click on "View HUPO Peptides".

Augment Library - Extracting - MSV000080544 Hits 1 ~ 30 out of 40258 Go to Go Export Filtered Results
Select columns

Filter	Protein	FDR	score	length	UniProt PE	neXtProt PE	HUPO Non-Overlapping Peptides	HUPO Peptides
1	sp|Q86YS7|C2CD5_HUMAN	0.000	1078.343	1000	1	1	66	View HUPO Peptides
2	sp|Q9Y6Y8|S23IP_HUMAN	0.000	1910.129	1000	1	1	72	View HUPO Peptides
3	sp|Q2VWA4|SKOR2_HUMAN	0.000	536.876	1001	1	1	20	View HUPO Peptides
4	sp|Q8N283|ANR35_HUMAN	0.000	362.596	1001	2	1	23	View HUPO Peptides
5	sp|O14983|AT2A1_HUMAN	0.000	449.944	1001	1	1	25	View HUPO Peptides
6	sp|Q7L7X3|TAOK1_HUMAN	0.000	943.023	1001	1	1	36	View HUPO Peptides
7	sp|Q13472|TOP3A_HUMAN	0.000	655.920	1001	1	1	45	View HUPO Peptides

This highlights all the peptides that are of sufficient length, do not differ by single amino acid variant to another peptide in the database, or are ambiguously identified for the given protein. To trace back the provenance information to all candidate replicate spectra in MassIVE-KB click on "All Candidate Spectra".

Note 2. All Precursor Observations in MassIVE-KB

The latest versions of the MassIVE-KB are available at http://massive.ucsd.edu/ProteoSAFe/static/massive-kb-libraries.jsp.

All provenance information is accessible for each MassIVE-KB in this link:

Download	Library Name	Description	Precursors	Peptides	Proteins
MGF | sptxt	Human HCD Spectral Library	This is the Human HCD spectral library. Containing over 2.1M precursors and drawing on over 30TB of MS/MS proteomics data.	2,122,892	1,088,645	19,610

To view all precursors in MassIVE-KB, click on this link:

View Library
[Library Variants]

This will list all precursors that were identified and included in MassIVE-KB. To query for a specific peptide, enter the peptide of interest in the "peptide" column. For example: NTSMEPAAETGKPPTVK charge 3.

For each entry, we can visualize the spectrum.

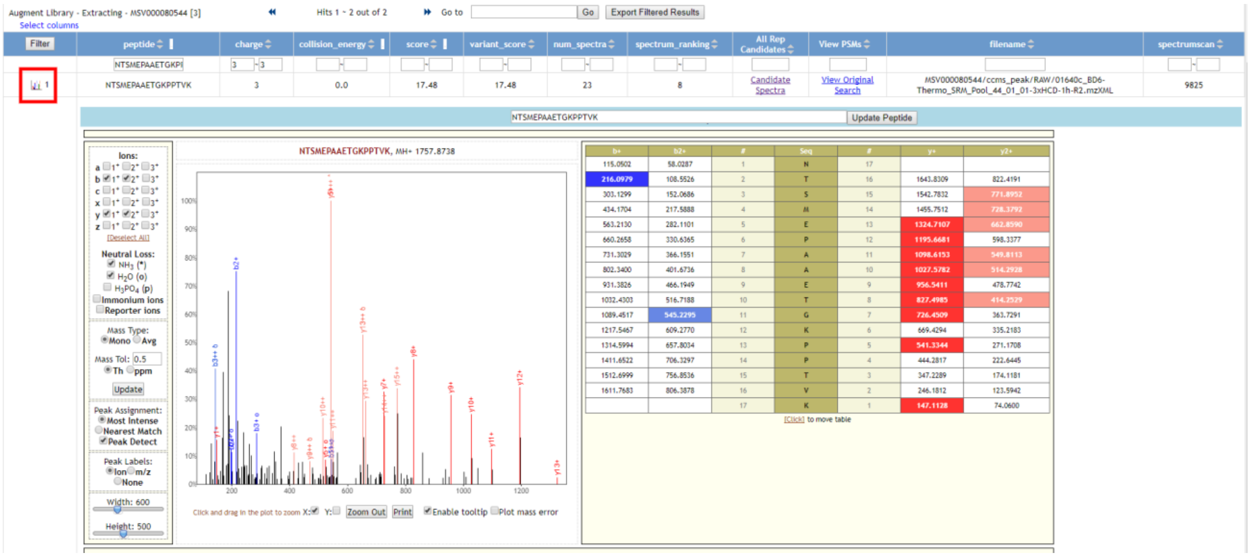

We can find all the spectra in MassIVE where this peptide was identified during MassIVE-KB construction by clicking the "Candidate Spectra" link.

Augment Library - Extracting - MSV000080544 [3] Hits 1 ~ 2 out of 2

Filter	peptide	charge	collision_energy	score	variant_score	num_spectra	spectrum_ranking	All Rep Candidates	View PSMs	filename	spectrumscan
	NTSMEPAAETGKPI	3 ~ 3									
1	NTSMEPAAETGKPPTVK	3	0.0	17.48	17.48	23	8	Candidate Spectra	View Original Search	MSV000080544/ccms_peak/RAW/01640c_BD6-Thermo_SRM_Pool_44_01_01-3xHCD-1h-R2.mzXML	9825
2	NTSMEPAAETGKPPTVKSPPTVK	3	0.0	18.08	21.07	20	7	Candidate Spectra	View Original Search	MSV000080544/ccms_peak/RAW/01866a_GA3-TUM_second_pool_105_02_01-3xHCD-1h-R1.mzXML	9653

This gives us the full list of spectra from the data that were in consideration for the representative spectra in MassIVE-KB.

Augment Library - Extracting - MSV000080544 [3] Hits 1 ~ 30 out of 43

#	annotation	score	charge	filename	scan	most_similar_score	ppm_error	explained_intensity
1	NTSMEPAAETGKPPTVK	16.677	3	MSV000080544/ccms_peak/RAW/01640c_BD6-Thermo_SRM_Pool_44_01_01-2xIT_2xHCD-1h-R2.mzXML	15118	0.71	0.10	0.76
2	NTSMEPAAETGKPPTVK	15.512	3	MSV000080544/ccms_peak/RAW/01640c_BD6-Thermo_SRM_Pool_44_01_01-2xIT_2xHCD-1h-R2.mzXML	14992	0.74	0.10	0.80
3	NTSMEPAAETGKPPTVK	17.482	3	MSV000080544/ccms_peak/RAW/01640c_BD6-Thermo_SRM_Pool_44_01_01-2xIT_2xHCD-1h-R2.mzXML	14931	0.73	0.11	0.81
4	NTSMEPAAETGKPPTVK	16.176	3	MSV000080544/ccms_peak/RAW/01640c_BD6-Thermo_SRM_Pool_44_01_01-2xIT_2xHCD-1h-R2.mzXML	14874	0.72	0.20	0.81
5	NTSMEPAAETGKPPTVK	15.439	3	MSV000080544/ccms_peak/RAW/01640c_BD6-Thermo_SRM_Pool_44_01_01-2xIT_2xHCD-1h-R2.mzXML	15057	0.74	0.01	0.78
6	NTSMEPAAETGKPPTVK	4.217	3	mwang87/data/Proteomics/PeptideLibraryCreation/Raw_Data/Bioplex_Summary/all_mzXML/j12178_C4orf17.mzXML	4395	0.52	0.01	0.66
7	NTSMEPAAETGKPPTVK	16.625	3	MSV000080544/ccms_peak/RAW/01640c_BD6-Thermo_SRM_Pool_44_01_01-3xHCD-1h-R2.mzXML	13119	0.74	0.01	0.70
8	NTSMEPAAETGKPPTVK	17.482	3	MSV000080544/ccms_peak/RAW/01640c_BD6-Thermo_SRM_Pool_44_01_01-3xHCD-1h-R2.mzXML	13009	0.79	0.22	0.75
9	NTSMEPAAETGKPPTVK	6.575	3	MSV000080826/ccms_peak/RAW/QE07594.mzML	5993	0.45	0.83	0.56
10	NTSMEPAAETGKPPTVK	17.482	3	MSV000080544/ccms_peak/RAW/01640c_BD6-Thermo_SRM_Pool_44_01_01-3xHCD-1h-R2.mzXML	13058	0.78	0.11	0.75
11	NTSMEPAAETGKPPTVK	16.697	3	MSV000080544/ccms_peak/RAW/01640c_BD6-Thermo_SRM_Pool_44_01_01-3xHCD-1h-R2.mzXML	12874	0.77	0.22	0.77
12	NTSMEPAAETGKPPTVK	17.482	3	MSV000080544/ccms_peak/RAW/01640c_BD6-Thermo_SRM_Pool_44_01_01-3xHCD-1h-R2.mzXML	12905	0.78	0.20	0.77
13	NTSMEPAAETGKPPTVK	16.208	3	MSV000080544/ccms_peak/RAW/01640c_BD6-Thermo_SRM_Pool_44_01_01-3xHCD-1h-R2.mzXML	13060	0.70	0.11	0.59
14	NTSMEPAAETGKPPTVK	16.823	3	MSV000080544/ccms_peak/RAW/01640c_BD6-Thermo_SRM_Pool_44_01_01-3xHCD-1h-R2.mzXML	13011	0.70	0.22	0.60
15	NTSMEPAAETGKPPTVK	16.360	3	MSV000080544/ccms_peak/RAW/01640c_BD6-Thermo_SRM_Pool_44_01_01-3xHCD-1h-R2.mzXML	12907	0.69	0.20	0.62
16	NTSMEPAAETGKPPTVK	16.823	3	MSV000080544/ccms_peak/RAW/01640c_BD6-Thermo_SRM_Pool_44_01_01-3xHCD-1h-R2.mzXML	12876	0.70	0.22	0.63
17	NTSMEPAAETGKPPTVK	15.873	3	MSV000080544/ccms_peak/RAW/01640c_BD6-Thermo_SRM_Pool_44_01_01-3xHCD-1h-R2.mzXML	13121	0.67	0.01	0.60
18	NTSMEPAAETGKPPTVK	15.459	3	mwang87/data/Proteomics/PeptideLibraryCreation/Raw_Data/Bioplex_Summary/all_mzXML/j12458_C4orf17.mzXML	4333	0.79	1.79	0.72
19	NTSMEPAAETGKPPTVK	16.535	3	MSV000080544/ccms_peak/RAW/01640c_BD6-Thermo_SRM_Pool_44_01_01-3xHCD-1h-R2.mzXML	13120	0.79	0.01	0.64

We can see the files where this precursor was found in. Specifically, this precursor was found in both the ProteomeTools Data (Blue Box) as well as in the Bioplex AP-MS data. Precursor was found in the pulldown of the C4orf17 gene, which corresponds to appropriate protein Q53FE4. We can further validate the fragmentation of the spectrum from the AP-MS data:

#	annotation	score	charge	filename	scan	most_similar_score	ppm_error
18	NTSMEPAAETGKPPTVK	15.459	3	mwang87/data/Proteomics/PeptideLibraryCreation/Raw_Data/Bioplex_Summary/all_mzXML/j12458_C4orf17.mzXML	4333	0.79	1.79

NTSMEPAAETGKPPTVK Update Peptide

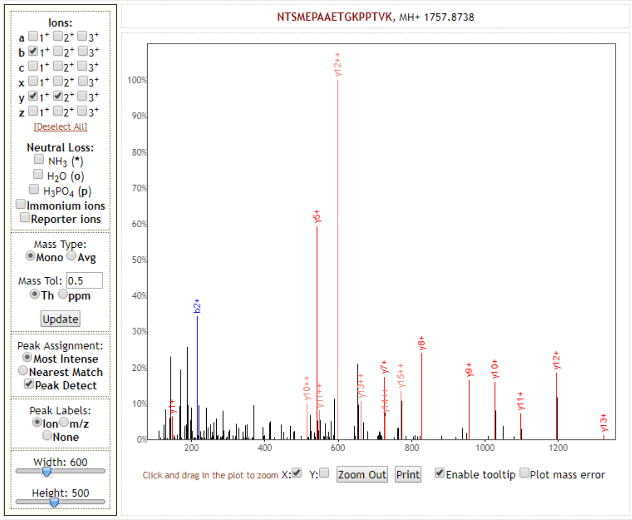

b+	#	Seq	#	y+	y2+
115.0502	1	N	17		
216.0979	2	T	16	1643.8309	822.4191
303.1299	3	S	15	1542.7832	771.8952
434.1704	4	M	14	1455.7512	728.3792
563.2130	5	E	13	1324.7107	662.8590
660.2658	6	P	12	1195.6681	598.3377
731.3029	7	A	11	1098.6153	549.8113
802.3400	8	A	10	1027.5782	514.2928
931.3826	9	E	9	956.5411	478.7742
1032.4303	10	T	8	827.4985	414.2529
1089.4517	11	G	7	726.4509	363.7291
1217.5467	12	K	6	669.4294	335.2183
1314.5994	13	P	5	541.3344	271.1708
1411.6522	14	P	4	444.2817	222.6445
1512.6999	15	T	3	347.2289	174.1181
1611.7683	16	V	2	246.1812	123.5942
	17	K	1	147.1128	74.0600

[Click] to move table

Against the fragmentation of one of the synthetic spectra:

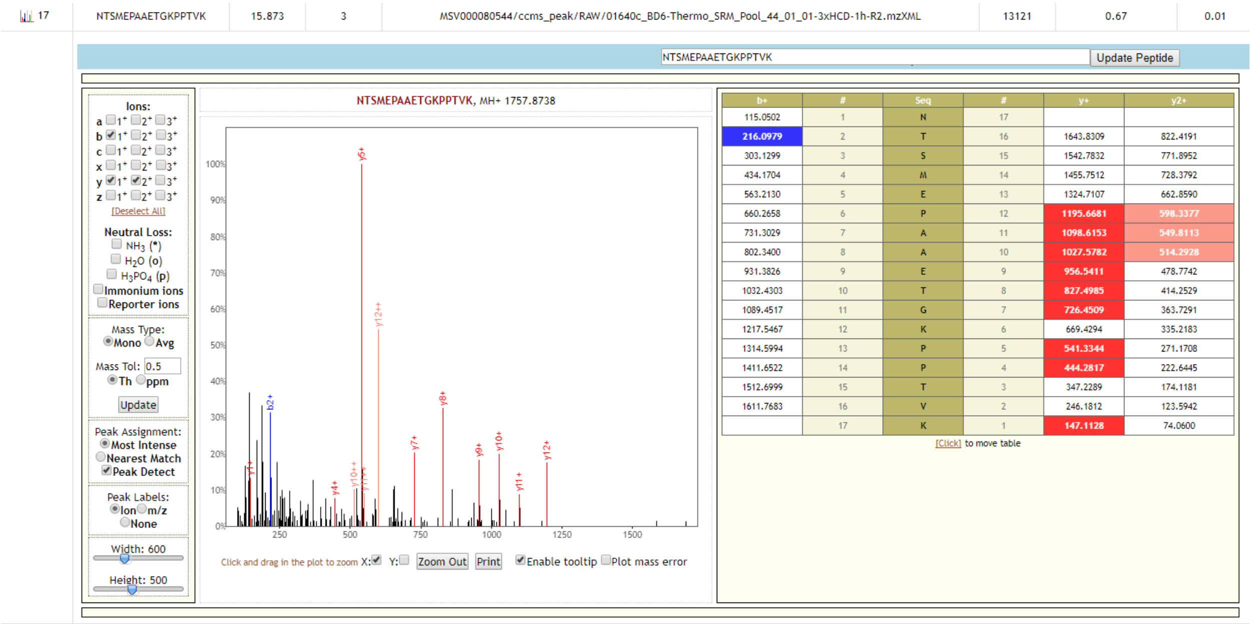

For each identified peptide spectrum match, we can trace back to the original database search that identified each spectrum by clicking on the “View Original Search”. This will take users back to the status page of the search performed by CCMS.

Note 3. Library Creation Provenance Record

The latest versions of the MassIVE-KB are available at http://massive.ucsd.edu/ProteoSAFe/static/massive-kb-libraries.jsp.

All provenance information is accessible for each MassIVE-KB in this link:

Download	Library Name	Description	Precursors	Peptides	Proteins
MGF | sptxt	Human HCD Spectral Library	This is the Human HCD spectral library. Containing over 2.1M precursors and drawing on over 30TB of MS/MS proteomics data.	2,122,892	1,088,645	19,610

The construction of MassIVE-KB occurred in several steps.

1. MS/MS data was searched by database tools
2. Identified MS/MS spectra were extracted
3. Extracted MS/MS were incorporated into MassIVE-KB

To view all database searches that were performed on input MassIVE-KB data click on “View All Search Tasks”.

Provenance
[All Candidate library spectra | View All Search Tasks | View All Library Augments | View All Library Spectrum Files]

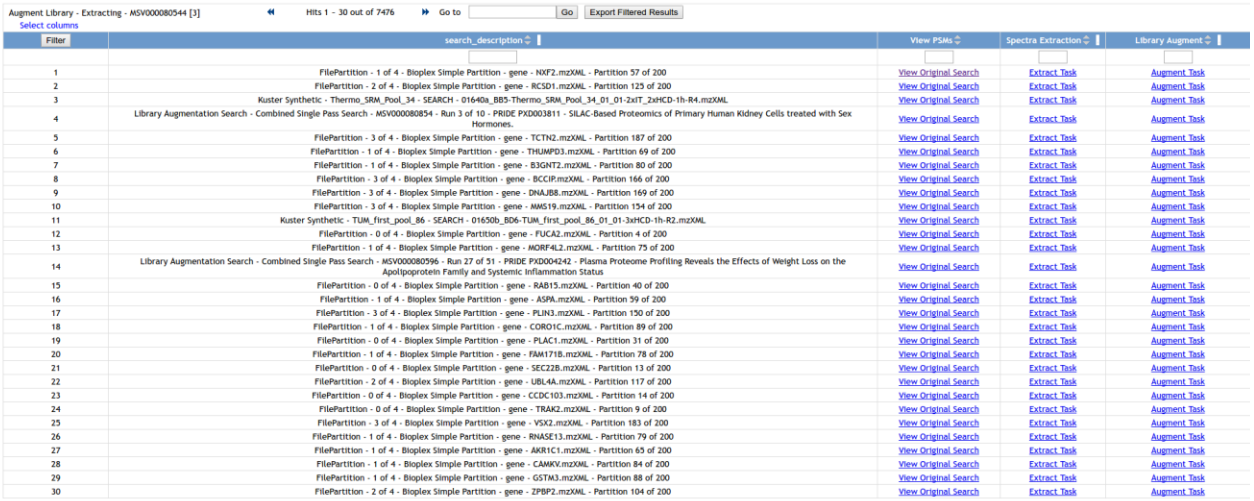

Augment Library - Extracting - MSV000080544 [3]   Hits 1 - 30 out of 7476   Go to [ ] Go   Export Filtered Results
Select columns

Filter	search_description	View PSMs	Spectra Extraction	Library Augment
1	FilePartition - 1 of 4 - Bioplex Simple Partition - gene - NXF2.mzXML - Partition 57 of 200	View Original Search	Extract Task	Augment Task
2	FilePartition - 2 of 4 - Bioplex Simple Partition - gene - RCSD1.mzXML - Partition 125 of 200	View Original Search	Extract Task	Augment Task
3	Kuster Synthetic - Thermo_SRM_Pool_34 - SEARCH - 01640a_BB5-Thermo_SRM_Pool_34_01_01-2xIT_2xHCD-1h-R4.mzXML	View Original Search	Extract Task	Augment Task
4	Library Augmentation Search - Combined Single Pass Search - MSV000080854 - Run 3 of 10 - PRIDE PXD003811 - SILAC-Based Proteomics of Primary Human Kidney Cells treated with Sex Hormones.	View Original Search	Extract Task	Augment Task
5	FilePartition - 3 of 4 - Bioplex Simple Partition - gene - TCTN2.mzXML - Partition 187 of 200	View Original Search	Extract Task	Augment Task
6	FilePartition - 1 of 4 - Bioplex Simple Partition - gene - THUMPD3.mzXML - Partition 69 of 200	View Original Search	Extract Task	Augment Task
7	FilePartition - 1 of 4 - Bioplex Simple Partition - gene - B3GNT2.mzXML - Partition 80 of 200	View Original Search	Extract Task	Augment Task
8	FilePartition - 3 of 4 - Bioplex Simple Partition - gene - BCCIP.mzXML - Partition 166 of 200	View Original Search	Extract Task	Augment Task
9	FilePartition - 3 of 4 - Bioplex Simple Partition - gene - DNAJB8.mzXML - Partition 169 of 200	View Original Search	Extract Task	Augment Task
10	FilePartition - 3 of 4 - Bioplex Simple Partition - gene - MMS19.mzXML - Partition 154 of 200	View Original Search	Extract Task	Augment Task
11	Kuster Synthetic - TUM_first_pool_86 - SEARCH - 01650b_BD6-TUM_first_pool_86_01_01-3xHCD-1h-R2.mzXML	View Original Search	Extract Task	Augment Task
12	FilePartition - 0 of 4 - Bioplex Simple Partition - gene - FUCA2.mzXML - Partition 4 of 200	View Original Search	Extract Task	Augment Task
13	FilePartition - 1 of 4 - Bioplex Simple Partition - gene - MORF4L2.mzXML - Partition 75 of 200	View Original Search	Extract Task	Augment Task
14	Library Augmentation Search - Combined Single Pass Search - MSV000080596 - Run 27 of 51 - PRIDE PXD004242 - Plasma Proteome Profiling Reveals the Effects of Weight Loss on the Apolipoprotein Family and Systemic Inflammation Status	View Original Search	Extract Task	Augment Task
15	FilePartition - 0 of 4 - Bioplex Simple Partition - gene - RAB15.mzXML - Partition 40 of 200	View Original Search	Extract Task	Augment Task
16	FilePartition - 1 of 4 - Bioplex Simple Partition - gene - ASPA.mzXML - Partition 59 of 200	View Original Search	Extract Task	Augment Task
17	FilePartition - 3 of 4 - Bioplex Simple Partition - gene - PLIN3.mzXML - Partition 150 of 200	View Original Search	Extract Task	Augment Task
18	FilePartition - 1 of 4 - Bioplex Simple Partition - gene - CORO1C.mzXML - Partition 89 of 200	View Original Search	Extract Task	Augment Task
19	FilePartition - 0 of 4 - Bioplex Simple Partition - gene - PLAC1.mzXML - Partition 31 of 200	View Original Search	Extract Task	Augment Task
20	FilePartition - 1 of 4 - Bioplex Simple Partition - gene - FAM171B.mzXML - Partition 78 of 200	View Original Search	Extract Task	Augment Task
21	FilePartition - 0 of 4 - Bioplex Simple Partition - gene - SEC22B.mzXML - Partition 13 of 200	View Original Search	Extract Task	Augment Task
22	FilePartition - 2 of 4 - Bioplex Simple Partition - gene - UBL4A.mzXML - Partition 117 of 200	View Original Search	Extract Task	Augment Task
23	FilePartition - 0 of 4 - Bioplex Simple Partition - gene - CCDC103.mzXML - Partition 14 of 200	View Original Search	Extract Task	Augment Task
24	FilePartition - 0 of 4 - Bioplex Simple Partition - gene - TRAK2.mzXML - Partition 9 of 200	View Original Search	Extract Task	Augment Task
25	FilePartition - 3 of 4 - Bioplex Simple Partition - gene - VSX2.mzXML - Partition 183 of 200	View Original Search	Extract Task	Augment Task
26	FilePartition - 1 of 4 - Bioplex Simple Partition - gene - RNASE13.mzXML - Partition 79 of 200	View Original Search	Extract Task	Augment Task
27	FilePartition - 1 of 4 - Bioplex Simple Partition - gene - AKR1C1.mzXML - Partition 65 of 200	View Original Search	Extract Task	Augment Task
28	FilePartition - 1 of 4 - Bioplex Simple Partition - gene - CAMKV.mzXML - Partition 84 of 200	View Original Search	Extract Task	Augment Task
29	FilePartition - 1 of 4 - Bioplex Simple Partition - gene - GSTM3.mzXML - Partition 88 of 200	View Original Search	Extract Task	Augment Task
30	FilePartition - 2 of 4 - Bioplex Simple Partition - gene - ZPBP2.mzXML - Partition 104 of 200	View Original Search	Extract Task	Augment Task

Clicking one of these search jobs, takes a user to the status page of the search task. Here users can click on the “Clone” button to visualize all selected parameters in the original analysis. Further users can rerun the identical analysis, facilitating reproducibility.

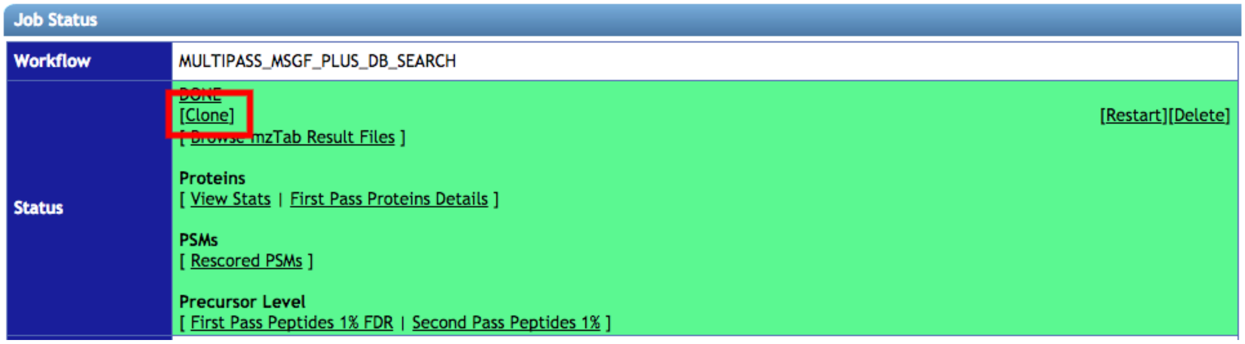

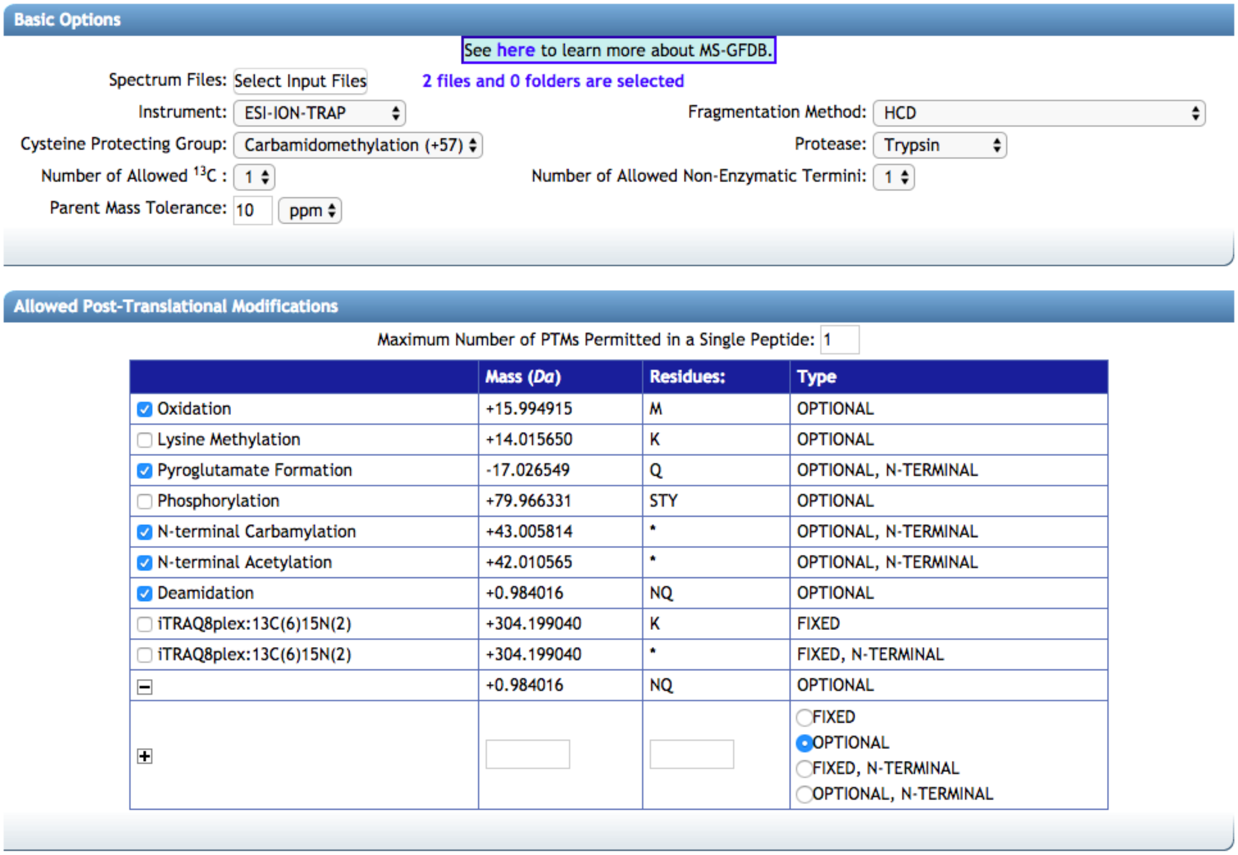

	Mass (Da)	Residues:	Type
☑ Oxidation	+15.994915	M	OPTIONAL
☐ Lysine Methylation	+14.015650	K	OPTIONAL
☑ Pyroglutamate Formation	-17.026549	Q	OPTIONAL, N-TERMINAL
☐ Phosphorylation	+79.966331	STY	OPTIONAL
☑ N-terminal Carbamylation	+43.005814	*	OPTIONAL, N-TERMINAL
☑ N-terminal Acetylation	+42.010565	*	OPTIONAL, N-TERMINAL
☑ Deamidation	+0.984016	NQ	OPTIONAL
☐ iTRAQ8plex:13C(6)15N(2)	+304.199040	K	FIXED
☐ iTRAQ8plex:13C(6)15N(2)	+304.199040	*	FIXED, N-TERMINAL
⊟	+0.984016	NQ	OPTIONAL
⊞			○ FIXED<br>◉ OPTIONAL<br>○ FIXED, N-TERMINAL<br>○ OPTIONAL, N-TERMINAL

Similarly, to examine all MS/MS public data files that were searched, click on "View All Library Spectrum Files".

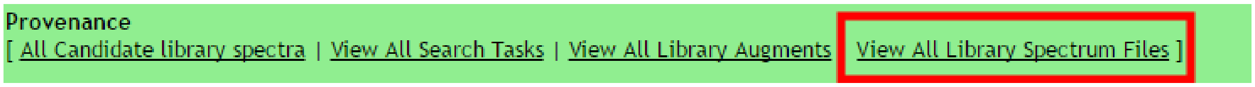

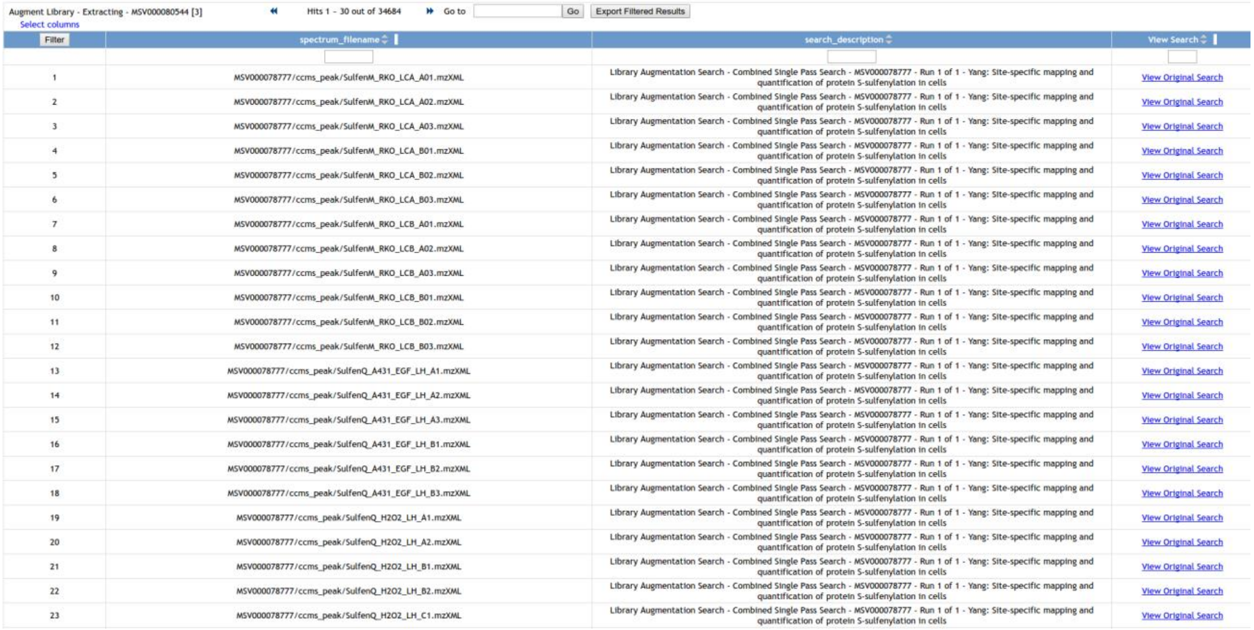

Filter	spectrum_filename	search_description	View Search
1	MSV000078777/ccms_peak/SulfenM_RKO_LCA_A01.mzXML	Library Augmentation Search - Combined Single Pass Search - MSV000078777 - Run 1 of 1 - Yang: Site-specific mapping and quantification of protein S-sulfenylation in cells	View Original Search
2	MSV000078777/ccms_peak/SulfenM_RKO_LCA_A02.mzXML	Library Augmentation Search - Combined Single Pass Search - MSV000078777 - Run 1 of 1 - Yang: Site-specific mapping and quantification of protein S-sulfenylation in cells	View Original Search
3	MSV000078777/ccms_peak/SulfenM_RKO_LCA_A03.mzXML	Library Augmentation Search - Combined Single Pass Search - MSV000078777 - Run 1 of 1 - Yang: Site-specific mapping and quantification of protein S-sulfenylation in cells	View Original Search
4	MSV000078777/ccms_peak/SulfenM_RKO_LCA_B01.mzXML	Library Augmentation Search - Combined Single Pass Search - MSV000078777 - Run 1 of 1 - Yang: Site-specific mapping and quantification of protein S-sulfenylation in cells	View Original Search
5	MSV000078777/ccms_peak/SulfenM_RKO_LCA_B02.mzXML	Library Augmentation Search - Combined Single Pass Search - MSV000078777 - Run 1 of 1 - Yang: Site-specific mapping and quantification of protein S-sulfenylation in cells	View Original Search
6	MSV000078777/ccms_peak/SulfenM_RKO_LCA_B03.mzXML	Library Augmentation Search - Combined Single Pass Search - MSV000078777 - Run 1 of 1 - Yang: Site-specific mapping and quantification of protein S-sulfenylation in cells	View Original Search
7	MSV000078777/ccms_peak/SulfenM_RKO_LCB_A01.mzXML	Library Augmentation Search - Combined Single Pass Search - MSV000078777 - Run 1 of 1 - Yang: Site-specific mapping and quantification of protein S-sulfenylation in cells	View Original Search
8	MSV000078777/ccms_peak/SulfenM_RKO_LCB_A02.mzXML	Library Augmentation Search - Combined Single Pass Search - MSV000078777 - Run 1 of 1 - Yang: Site-specific mapping and quantification of protein S-sulfenylation in cells	View Original Search
9	MSV000078777/ccms_peak/SulfenM_RKO_LCB_A03.mzXML	Library Augmentation Search - Combined Single Pass Search - MSV000078777 - Run 1 of 1 - Yang: Site-specific mapping and quantification of protein S-sulfenylation in cells	View Original Search
10	MSV000078777/ccms_peak/SulfenM_RKO_LCB_B01.mzXML	Library Augmentation Search - Combined Single Pass Search - MSV000078777 - Run 1 of 1 - Yang: Site-specific mapping and quantification of protein S-sulfenylation in cells	View Original Search
11	MSV000078777/ccms_peak/SulfenM_RKO_LCB_B02.mzXML	Library Augmentation Search - Combined Single Pass Search - MSV000078777 - Run 1 of 1 - Yang: Site-specific mapping and quantification of protein S-sulfenylation in cells	View Original Search
12	MSV000078777/ccms_peak/SulfenM_RKO_LCB_B03.mzXML	Library Augmentation Search - Combined Single Pass Search - MSV000078777 - Run 1 of 1 - Yang: Site-specific mapping and quantification of protein S-sulfenylation in cells	View Original Search
13	MSV000078777/ccms_peak/SulfenQ_A431_EGF_LH_A1.mzXML	Library Augmentation Search - Combined Single Pass Search - MSV000078777 - Run 1 of 1 - Yang: Site-specific mapping and quantification of protein S-sulfenylation in cells	View Original Search
14	MSV000078777/ccms_peak/SulfenQ_A431_EGF_LH_A2.mzXML	Library Augmentation Search - Combined Single Pass Search - MSV000078777 - Run 1 of 1 - Yang: Site-specific mapping and quantification of protein S-sulfenylation in cells	View Original Search
15	MSV000078777/ccms_peak/SulfenQ_A431_EGF_LH_A3.mzXML	Library Augmentation Search - Combined Single Pass Search - MSV000078777 - Run 1 of 1 - Yang: Site-specific mapping and quantification of protein S-sulfenylation in cells	View Original Search
16	MSV000078777/ccms_peak/SulfenQ_A431_EGF_LH_B1.mzXML	Library Augmentation Search - Combined Single Pass Search - MSV000078777 - Run 1 of 1 - Yang: Site-specific mapping and quantification of protein S-sulfenylation in cells	View Original Search
17	MSV000078777/ccms_peak/SulfenQ_A431_EGF_LH_B2.mzXML	Library Augmentation Search - Combined Single Pass Search - MSV000078777 - Run 1 of 1 - Yang: Site-specific mapping and quantification of protein S-sulfenylation in cells	View Original Search
18	MSV000078777/ccms_peak/SulfenQ_A431_EGF_LH_B3.mzXML	Library Augmentation Search - Combined Single Pass Search - MSV000078777 - Run 1 of 1 - Yang: Site-specific mapping and quantification of protein S-sulfenylation in cells	View Original Search
19	MSV000078777/ccms_peak/SulfenQ_H2O2_LH_A1.mzXML	Library Augmentation Search - Combined Single Pass Search - MSV000078777 - Run 1 of 1 - Yang: Site-specific mapping and quantification of protein S-sulfenylation in cells	View Original Search
20	MSV000078777/ccms_peak/SulfenQ_H2O2_LH_A2.mzXML	Library Augmentation Search - Combined Single Pass Search - MSV000078777 - Run 1 of 1 - Yang: Site-specific mapping and quantification of protein S-sulfenylation in cells	View Original Search
21	MSV000078777/ccms_peak/SulfenQ_H2O2_LH_B1.mzXML	Library Augmentation Search - Combined Single Pass Search - MSV000078777 - Run 1 of 1 - Yang: Site-specific mapping and quantification of protein S-sulfenylation in cells	View Original Search
22	MSV000078777/ccms_peak/SulfenQ_H2O2_LH_B2.mzXML	Library Augmentation Search - Combined Single Pass Search - MSV000078777 - Run 1 of 1 - Yang: Site-specific mapping and quantification of protein S-sulfenylation in cells	View Original Search
23	MSV000078777/ccms_peak/SulfenQ_H2O2_LH_C1.mzXML	Library Augmentation Search - Combined Single Pass Search - MSV000078777 - Run 1 of 1 - Yang: Site-specific mapping and quantification of protein S-sulfenylation in cells	View Original Search

To view all MS/MS extractions run on data and iterative augments to MassIVE-KB click on “View All Library Augments”.

Provenance
[All Candidate library spectra | View All Search Tasks | View All Library Augments | View All Library Spectrum Files]

This view shows that MassIVE-KB was augmented 10 iterations from 10 extractions of MS/MS data. To examine each augmentation and view a snapshot of MassIVE-KB at a given time point in the past, click on the respective “Library Augment”.

Augment Library - Extracting - MSV000080544 [3]   Hits 1 - 10 out of 10   Go to   Go   Export Filtered Results
Select columns

Filter	Spectra Extraction	Library Augment	timestamp
1	Extract Task	Augment Task	2017-02-13 11:59:33.0
2	Extract Task	Augment Task	2017-05-19 16:39:08.0
3	Extract Task	Augment Task	2017-01-28 17:54:44.0
4	Extract Task	Augment Task	2017-04-07 10:10:37.0
5	Extract Task	Augment Task	2017-04-05 11:45:02.0
6	Extract Task	Augment Task	2017-02-11 19:02:25.0
7	Extract Task	Augment Task	2017-04-08 11:32:36.0
8	Extract Task	Augment Task	2017-01-02 17:26:50.0
9	Extract Task	Augment Task	2017-04-12 23:44:38.0
10	Extract Task	Augment Task	2017-02-22 21:59:48.0

Finally, to download full provenance record of all candidate replicate MS/MS spectra for the library, click on the “All Candidate library spectra” link.

Provenance
All Candidate library spectra | View All Search Tasks | View All Library Augments | View All Library Spectrum Files]

This view contains all top 100 MS/MS replicate spectra per library precursor along with their provenance information, e.g. original database search scores, charge, public dataset file path, scan number, ProteoSAFe search task, explained intensity, similarity to representative, and library creation tasks that brought it into MassIVE-KB. To download in bulk, click the download button on top:

Back to main page   Back to status page   Collapse all   Download
